# In situ analysis of vascular structures in fractured *Tyrannosaurus rex* rib

**DOI:** 10.1038/s41598-025-06981-z

**Published:** 2025-07-04

**Authors:** Jerit L. Mitchell, Mauricio Barbi, Ryan C. McKellar, Monica Cliveti, Ian M. Coulson

**Affiliations:** 1 Department of Physics, University of Regina, Regina, Saskatchewan S4S 0A2 Canada; 2 Royal Saskatchewan Museum, 2445 Albert St., Regina, S4P 4W7 Saskatchewan Canada; 3 Department of Biology, University of Regina, Regina, S4S 0A2 Saskatchewan Canada; 4 Department of Earth Sciences, University of Regina, Regina, Saskatchewan, S4S 0A2 Canada

**Keywords:** Dinosaur, Synchrotron, Soft tissue, Blood vessel, Angiogenesis, Pathology, Iron, Palaeoecology, Palaeontology

## Abstract

Soft tissue preservation in fossils has become a popular focus of paleontology research due to easier access to sensitive probes like synchrotron radiation, allowing more detailed analysis of specimens. Although uncommon, reports exist on vascular preservation in dinosaurs, generally as remnants of Haversian canals. However, combined 3D morphological and chemical analysis of large angiogenic dinosaur blood vessels has not been reported before. Here we show characterization of a network of large vessel-like structures in a rib from “RSKM P2523.8” (Royal Saskatchewan Museum), an exceptionally robust *Tyrannosaurus rex* found in the Late Cretaceous Frenchman Formation, Saskatchewan, Canada. Using Synchrotron Micro-Computed Tomography these structures can be visualized in situ within the bone and matched to chemical microprobing from Synchrotron X-ray Fluorescence and X-ray Absorption Near Edge Structure. Combined with conventional optical and electron microscopy, we show the vessel-like structures are composed of pyrite partially oxidized to goethite or hematite, preserved in two distinct layers as permineralized casts. Although no original soft tissues were able to be recovered using the current suite of techniques, the structures’ morphology and sole presence in a fractured area of the rib suggest angiogenic origin. Bone healing and regrowth may offer a promising target for future multi-technique soft tissue experiments analyzing dinosaur healing potential.

## Introduction

Investigations of “life-like” soft tissue preservation in fossils have become more commonplace, particularly in the Cretaceous period^1^, through increased adoption of high-intensity synchrotron radiation techniques^2^. One common type of fossil with reports of soft tissue is bone, where preserved components such as osteocytes, chondrocytes, nerves, blood vessels and collagen sheets have been described, and recently reviewed^3,4^. Older publications have claimed the discovery of blood cells in dinosaur blood vessels^5,6^, but these have also been reinterpreted as diagenetic alterations^7^. In particular, the preservation of soft and pliable blood vessels has been reported in various dinosaurs (including *Tyrannosaurus rex* (*T. rex*))^8–14^, predominantly through the pioneering work of a few research groups. A recent study^14^ used nano-CT along with other imaging techniques to detect pliable-type vessels of multiple dinosaur taxa, and compared the morphology to an extant animal. Chemical analysis of these structures has found the preservation associated with iron in the 3+ state, which is thought to be of potentially endogenous origin^4,8,15^. These vessels have even been reported to be associated with the preservation of collagen proteins in some cases^8,14,16,17^. The most common interpretation is that such vessels have been preserved via Fenton reactions, which make use of iron liberated from blood hemoglobin to produce cross links in protein structures, stabilizing them over the long term^3,8^. All of the mineralized vessels preserved thus far have been reported in the form of remains of Haversian canals, with other types of vessels being unrepresented.

Pathologies, such as fractures, can lead to a rapid increase in vascular activity, known as angiogenesis. New vessels branch off of old vessels from the normal bone and expand towards the fracture/callus in order to bring nutrients to facilitate the healing of the injury^18^. The vessels thus produced are generally larger than those found in the Haversian canals of osteons as they supply a larger volume of blood and can include contributions from the medullary (marrow) cavity, cortical, and periosteal (outer bone coating) vessels^19^. Many studies of pathological dinosaur bones rely on histological analysis or 2D CT slices produced via table top systems^20–24^, but synchrotron studies have been performed in analyses of pathological dinosaur bone^25^. Also, 3D CT reconstruction of remnant vascularity in dinosaur skulls and dentaries, including *T. rex*, has been performed^26–29^. However, high resolution (on the order of microns) in situ (analysis of structures still within original bone) combined 3D morphology and complementary chemical analyses of dinosaur healing angiogenesis is absent in current literature. Such a detailed study of bone fracture microstructure could provide new information on bone healing and remodeling in dinosaurs.

The study here focuses on “RSKM P2523.8”, a *Tyrannosaurus rex* specimen which is part of the collection at the Royal Saskatchewan Museum. RSKM P2523.8 is currently estimated to be the largest ever recovered *T. rex* specimen and it was found in 1991 near Eastend, Saskatchewan, Canada. With 65% of the skeleton recovered from the Late (Upper) Cretaceous Frenchman Formation ($\sim$67 Ma), RSKM P2523.8 is also one of the most complete *T. rex* specimens ever recovered^30^. The surrounding rocks are composed of sandstone with sporadic ironstone concretions, and much of the evidence for predicting the depositional environment of the specimen indicates a low energy river or floodplain. For this reason the individual is thought to have died close to the final burial site; however, highly oxidizing conditions are reported in the deposit, suggesting that the body was left exposed for some extended period of time before burial^31,32^. The site RSKM P2523.8 was found in is also abundant in plant fossils, which is unusual as plants are commonly preserved best in acidic and reducing conditions which are typically less conducive to bone preservation^32^. The sediment conditions and pore water chemistry likely are critical for the quantity and quality of preservation of RSKM P2523.8^33^. This tyrannosaur skeleton features many pathologies including ones on the tail, skull, and ribs. A recovered dorsal rib head relevant to this study features a large callus at the point of an injury induced fracture, and appears incompletely healed^30^.

In this pilot study, we analyze vessel-like structures within the fractured rib head from RSKM P2523.8 using various techniques, both from an in situ morphological perspective as well as a qualitative and quantitative look at the chemical composition. The availability of multiple beamlines at the Canadian Light Source^34^ synchrotron facility for CT imaging and chemical analysis provide an opportunity to characterize possible vessel structures in unmatched resolution. Techniques used in the study include those aided by synchrotron radiation (Micro-Computed Tomography ($\upmu$CT), X-ray Absorption Near Edge Structure (XANES), X-ray Fluorescence (XRF) spectroscopy and mapping), as well as more traditional techniques such as optical and reflected light microscopy, and Scanning Electron Microscopy (SEM), including Back Scattered Electron imaging (BSE) as well as Energy Dispersive Spectroscopy (EDS). Lastly, there will be a discussion on the utility of synchrotron in situ techniques for fossil bone analysis, the preservation pathways of the vessel-like structures observed in the RSKM P2523.8 bone as compared to other reports, and the possible impact of the results on future soft tissue studies relating to pathologies and bone healing.

## Methods

### Microscopy

Microscopy and petrographic analysis were performed at the Royal Saskatchewan Museum and University of Regina. Histologic features and diagenetic changes were studied in two thin sections of bone observed under plane and cross-polarized light, with a Nikon OptiPhot petrographic microscope. At times, an accessory (gypsum; 1$\lambda$) plate was inserted into the light path to assist in the measurement of a variety of optical properties. Pictures of the thin sections were taken using a Leica 3 camera attachment. The thin sections were also studied with reflective light methods to aid in the determination of the composition of a variety of infilling minerals. Histological terminology and definitions generally follow those of National Cancer Institute, US. Petrographic analysis allowed for determining the bulk mineral composition of the studied rib bone, identifying the composition of the infilling minerals, and the estimating of the taphonomic process suffered by the bone. Taphonomic features, such as cracking, fracturing, deformation and permineralization were assessed as part of the petrographic analysis.

### CT

Propagation Phase-Contrast Synchrotron Radiation Microtomography (PPC-SR$\upmu$CT) scans of the rib were performed at the Canadian Light Source (CLS) synchrotron light facility, using the Biomedical Imaging and Therapy Facility (BMIT-ID) beamline^34^. A beam energy of 80 keV was used with a sample-to-detector distance of 1 m. 6000 projections were taken over 180$^{\circ }$ at an exposure time of 1.4 ms each in order to produce the tomographic slices. The original detector resolution of 8.91 $\upmu$m was binned in $2 \times 2$ to decrease acquisition time, resulting a slice resolution of 17.82 $\upmu$m. Reconstruction of the raw projection images was performed using *tofu* image processing and tomography reconstruction toolkit^35^ by beamline scientists at the CLS. Fiji^36^ (ImageJ) was used to prepare the CT slices for 3D segmentation via stacking of images, cropping, and contrast enhancement of images. 3D rendering and segmentation of 3D models was performed using Dragonfly Pro 2022.1^37^.

For the initial CT scan, a special absorption homogenization technique is used^38^. As the energy and flux of the X-rays used is very high, photons passing through the thinner areas of the large bone will over saturate the X-ray detector, such that the flux must be reduced. To solve this, the sample is placed in a cylinder with beads of a similar density along with a u-shaped profiler ahead of the sample. This effectively normalizes the absorption across the field of view such that the flux can be increased to improve the contrast and signal-to-noise ratio for the scan.

### XRF and XANES

Initial chemical analysis measurements were performed at the Soft X-ray Micro characterization Beamline (SXRMB)^34^ at the CLS. The energy range of the beamline (1.7–10 keV) allowed elements of silicon through iron to be probed effectively. $\upmu$XRF spectra were recorded on a grid of points in order to create chemical maps for elements of interest. The pixel resolution of the maps are 10 $\upmu$m. Fiji was used to create multi-element RGB maps. XANES micro-probe measurements were also performed on concentration “hot spots” found from the chemical maps. Athena, part of the Demeter^39^ spectroscopic analysis platform, was used to normalise the X-ray absorption data and reference spectra, as well as perform Linear Combination Fitting (LCF). LCF for the iron K-edge was performed using ten references, selecting the best fit of up to four references using combinatorics.

Further XRF mapping was performed at the Very sensitive Elemental and Structural Probe Employing Radiation from a Synchrotron (VESPERS)^34^ beamline at the CLS. This provided access to higher energy elements beyond iron, such as Sr and Y, as well as a better spot resolution of 5 $\upmu$m. The raw spectral data were deadtime and background corrected, and normalized using CERN ROOT^40^ macros in order to produce the maps and cuts on the data.

### SEM

Scanning Electron Microscopy (SEM) analyses were performed at the University of Regina, using the Faculty of Science’s electron microbeam facility. It utilizes a Tescan Vega 3 microscope fitted with an EDAX-Apex EDS system. Cross-sections of rib bone were carbon coated before they were imaged under SEM to prevent charging. Back scattered images show areas of higher atomic number material in lighter shades. EDS measurements as well as chemical mapping were obtained. The electron beam was accelerated to 10 KeV, with working distances of between 10 and 20 mm. EDS allows emission mapping of low atomic number elements such as carbon and oxygen (where it is most sensitive). The final spectra were visualized in CERN ROOT analysis framework.

## Results

### Sample overview

**Fig. 1:**
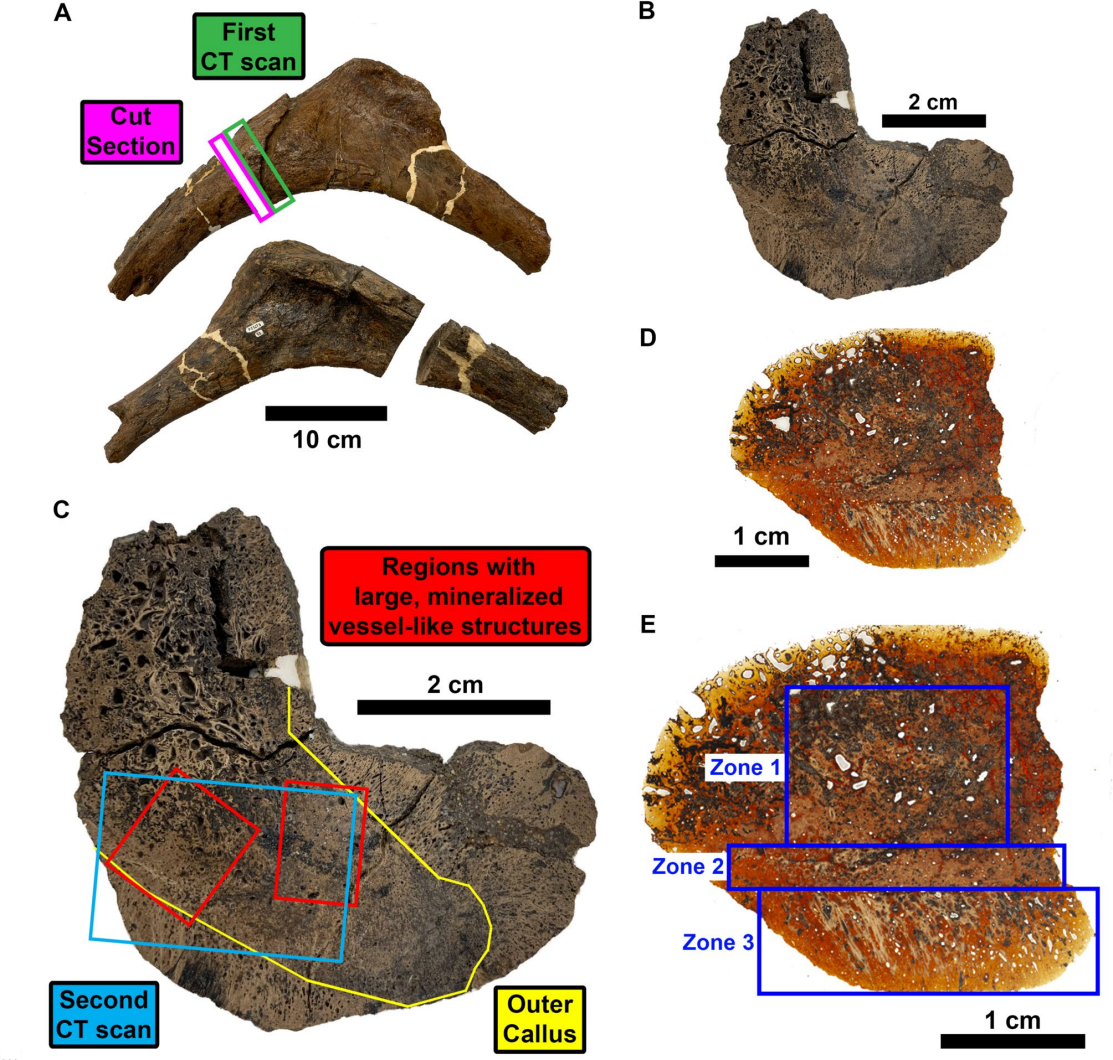
Photographs of the pathogenic rib from RSKM P2523.8. (**A**) Opposing views of the rib head from RSKM P2523.8. Locations on the callused area on the rib shaft that were chosen for initial CT scanning at BMIT (green box) as well as cut into a bone section (magenta box) are labeled. (**B**) Cross-sectional view of the cut bone billet section from (**A**). (**C**) Labelled version of billet of (**B**) with location of fracture induced bone growth (outside yellow line), further CT scanning (blue box), and general regions of the found mineralized vessel-like structures (red boxes). (**D**) Thin section cut from the billet of (**B**). (**E**) Labeled version of (**D**) with notable separate petrographic zones of bone growth.

The fractured rib from RSKM P2523.8 was chosen for exploratory analysis using $\upmu$CT in order to perform virtual histology (Fig. 1A). The rib features a deep healed fracture on the proximal shaft, near the head of the rib. An initial 1 cm thick, 9 $\upmu$m voxel, cross-sectional $\upmu$CT scan was performed on a location beside the fracture. In order to perform chemical analysis inside the bone, a $\sim$6 mm thick section was cut out of the callus to expose surfaces for chemical observations (Fig. 1B). This cut section or billet of bone resides directly adjacent to the first CT scan, connecting the two data sets. The callus that delineates primary bone growth due to the injury can be seen in the photographs (Fig. 1C). Further $\upmu$CT scanning was performed on the cut section. A second CT scan was performed in the region directly adjacent to where vessel-like structures from the first CT scan were found, as will be discussed in the next section. In relation to the rib cross section, these vessel-like structures in the bone can be found in the areas that are flanking the new bone growth areas^41^.

Thin sections of the bone were produced after CT analysis via polishing of the central section of the rib billet and from another billet cut in the same region labeled in (Fig. 1B). The analyzed thin sections of the bone (Fig. 1D,E) show a heterogeneous bone structure, with three different areas of the compact bone being identified.

### Petrography

Petrographically, the thin section examinations show a relative uniform fluorapatite mineralogical composition that is randomly overprinted by Fe-rich minerals. The Haversian canals, pores and older generation of fractures are coated to various degrees with Fe-rich minerals, from a thin coat to completely infilled. A few of the pores and the Haversian canals are filled in with gypsum or anhydrite. Petrographic zones on the rib section are as follows (Fig. 1E): (1) crushed Haversian structure; (2) well-developed Haversian structure with primary and secondary osteons; and (3) callus zone.

**Fig. 2:**
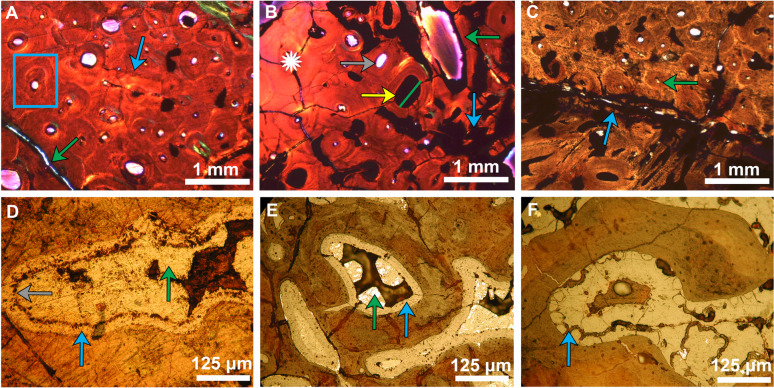
Petrographic analysis of pathogenic rib thin section from RSKM P2523.8. (**A**) Image in XPL, with the gypsum plate in, showing the Haversian system with calcified primary and secondary osteons showing evidence of internal bone remodelling in Zone 2 (Blue Square as an example of secondary osteon). Compared to the other regions of the bone, this Zone is almost pristine, with minimal overprinting by Fe-rich minerals. The blue arrow is pointing at a line of arrested growth (LAG). The younger generation of fracture (green arrow) is thinner; it is thinly coated with Fe-rich minerals and the center is either clear or infilled by silica. (**B**) Image in XPL, with the gypsum plate in, showing a compact bone structure with Haversian system showing the calcified osteons made of concentric lamellae and the Haversian canals that used to host the blood vessels and nerves (e.g. yellow arrow). Some of the Haversian canals are completely infilled by Fe-rich minerals, as in the case of the osteon in the centre of the image (yellow arrow; green line shows diameter of 0.64 mm); some of the canals are just coated in Fe-rich minerals (green arrow), and some are clear of any minerals (gray arrow). A radial fracture system, probably diagenetic related, is marked with a star on the left side of the slide. The blue arrow, on the right side of the slide points at an increase in Fe-rich minerals. (**C**) Image in PPL showing Zones 2 and 3. Zone 2, in the upper part of the picture, with well-developed Haversian system (i.e. compact bone of green arrow). Zone 3, in the lower part of the picture, shows an oblique cut through the callus zone. A fracture coated with Fe-rich minerals and reactivated by the diagenesis that separates the two zones (blue arrow). The osteocytes were active at the time of death. (**D**, **E**) Reflective microscopy images (from Zone 1) showing the two generations of pyrite separated by a layer of rust (gray layer). First generation is fine-grained pyrite (blue arrow); second generation is crystalline (green arrow). First generation has been almost completely oxidized to hematite; the second generation is in the process of turning into hematite. (**F**) Reflective light microscopy image showing that the first generation pyrite is botryoidal (blue arrow).

Zone 1, edge of medullar bone (Fig. 2D–F, Supplementary Fig. S1A–C) is located relatively centrally and is a zone of intense reworking and remodeling of the bone as the osteons and the Haversian canals are crushed. The original mineralogical composition is heavily overprinted by Fe-rich mineralization (Supplementary Fig. S1A,B, green arrows). The Haversian canals are observed to be mostly infilled and they record at least two episodes of mineral precipitation (Fig. 2D,E).

Zone 2, outer edge of cortical bone (Fig. 2A,B, Supplementary Fig. S1D) shows compact bone with closely packed Haversian systems. In this zone, there are well-developed primary and secondary osteons with calcified lamellae, with Haversian canals that can vary from clear/open to completely infilled with Fe-rich minerals, infilled canaliculi and lacunae, and preserved Volkmann’s canals (Fig. 2B). The diameters of the Haversian canals range between 0.23 and 0.6 mm, while the Volkmann’s canals measure between 0.05 and 0.25 mm. The overprinting with Fe-rich minerals is limited compared to the previous zone. The internal remodelling of the bone is evident by the presence of both primary and secondary osteons (Fig. 2A). The fracture system defined in this zone indicates at least two generations of fractures. The older generation consists of wider fractures that are almost or completely infilled with Fe-rich minerals (Fig. 2B, blue arrow). The younger generation is thinner and in a radial pattern (Fig. 2B, star), and in most cases free of mineral precipitates indicating a late diagenetic feature.

Zone 3, callus (Fig. 2C, Supplementary Fig. S1E) has a well-defined boundary atop Zone 2, where the periosteal surface existed before the callus formed. The distinction between the zones is visible in this thin section, due to the presence of a fracture that follows the boundary layer (Fig. 2C, blue arrow), but also by the fact that the bone changed growth direction, as indicated by the oblique cut through the bone structures. The Fe-rich minerals are limited to lining the walls of the Haversian canals. The preservation of this zone points to the fact that the dinosaur died within months of suffering the bone fracture as the healing process has started already, but it did not reach completion.

Reflected light microscopy (Fig. 2D–F, Supplementary Fig. S1C,D) was used initially to determine the mineralogy of the coating of the Haversian canals, but it proved extremely useful in understanding the diagenesis and the taphonomic processes that affected the bone. The microscopy studies showed that there were at least two distinct generations of pyrite. The first generation was a very fine or botryoidal pyrite that coated almost every available pore space (Fig. 2D–F, blue arrows). The second generation appears to be more concentrated in Zone 1, the medullary bone with larger porosity, and is coarser grained with pyrite actually forming individual crystals (Fig. 2D,E, green arrows). The two generations of pyrite are separated by a layer of “rust” minerals (Fig. 2D, gray arrow). Both generations of pyrite appear to have been partially oxidized to hematite. Some other minerals are associated with the two identified minerals, possibly galena, pyrrhotite, and magnetite, but more quantitative work is needed to confirm their presence (XRF and XANES analysis in ensuing sections).

### 3D tomography

**Fig. 3:**
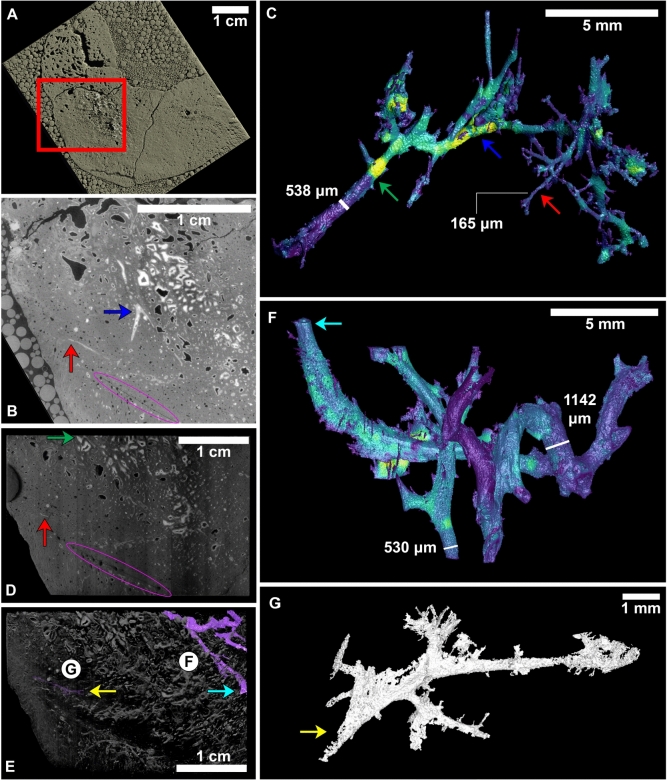
In situ SR-CT analysis of vessel-like structures in pathogenic rib of RSKM P2523.8 at BMIT-ID beamline. (**A**–**C**) Original CT scan as shown in Fig. 1A. (**A**) 3D threshold rendering of the exterior of the cross sectional scan. The location of the found vascular-type structures is given by the red box. Glass beads seen outside of the sample are part of the photon homogenization technique used. (**B**) A single tomographic slice, zooming into the box of (**A**), taken from the middle of the cross section. A notable high density structure arises (tunnel-like bright pixels). The magenta oval indicates the line separating the normal bone from the callus bone. (**C**) 3D thickness mesh rendering of the high density structure represented by (**B**). Brighter areas are mineralized, while darker areas are more hollow. The red and blue arrows of (**B**) and (**C**) show corresponding points in 2D and 3D visualization. (**D**–**G**) Second CT scan as shown in Fig. 1C. (**D**) 3D threshold render of the second scan, showing the exposed surface. The red arrow here indicates the specific location of the structure that was probed for subsequent chemical analysis, which is the same location as the first CT scan (**B**, **C**). The part of the vessel corresponding to the green arrow of (**C**) has been exposed here. (**E**) 3D threshold render with removal of lower density voxels to show location of more found vascular-type structures. (**F**) 3D thickness mesh of a larger tubular structure found closer to the center of the bone, with corresponding cyan arrow from (**E**). (**G**) 3D mesh render of a structure similar in morphology to the first CT scan (**C**), with corresponding yellow arrow from (**E**).

Initial analysis of the first CT scan showed unexpected high density structures bridging multiple zones within the bone (Fig. 3A). A long, meandering, high density structure is found running from the middle of the bone towards the outside of the bone (Fig. 3B). This structure runs orthogonal to the osteons and travels from the cancelous area of the bone (Zone 1) to the outer edge (of Zone 2), with new bone growth (marked with magenta oval) produced from the fracture. Based upon threshold rendering, a 3D segmentation of the structure could be created (Fig. 3C). The structure varies from 100 $\upmu$m to 500 $\upmu$m in diameter. Sections of the structure are completely infilled with high-density material (brighter areas), while other areas are hollow (darker areas). Based on these characteristics and the way the structure branches out, it resembles the morphological features of blood vessels in bone^19^.

A second CT was performed on the section of cut rib (Fig. 3D). A small part of the branching tubular structure (Fig. 3B–D, shown with a red arrow) is exposed on the surface of the cut section. This section of the tubular structure was chosen to be probed for additional chemical analyses. More vascular-like structures were able to be extracted out of this scan (Fig. 3E). Large vessel-like structures, $\sim$1 mm in diameter, are located by the callus on the other side of the bone (Fig. 3F). New vessel-like structures that are similar in size and position to those found in the first CT scan are also present in the second scan (Fig. 3G).

The structures discussed above appear to extend and network much farther and more elaborately than what is shown in Fig. 3, however, they are not infilled and therefore are much more difficult to segment out. Another CT scan was performed on the RSKM P2523.8 rib, on a section of normal bone 5 cm away from the fracture; however, 3D reconstruction of this section of the rib showed no evidence of similar high-density vessel-like structures. Since the observed vessel-like structures are not found in healthy parts of the bone, their presence in the pathological rib section suggest such vascular activity could be the result of healing angiogenesis. The 3D vascular activity is similar to CT experiments in mice that experienced a fracture^42^. All $\upmu$CT data produced in the analysis of this section, including slice data and 3D models, are available at MorphoSource^43^.

### 2D imaging

**Fig. 4:**
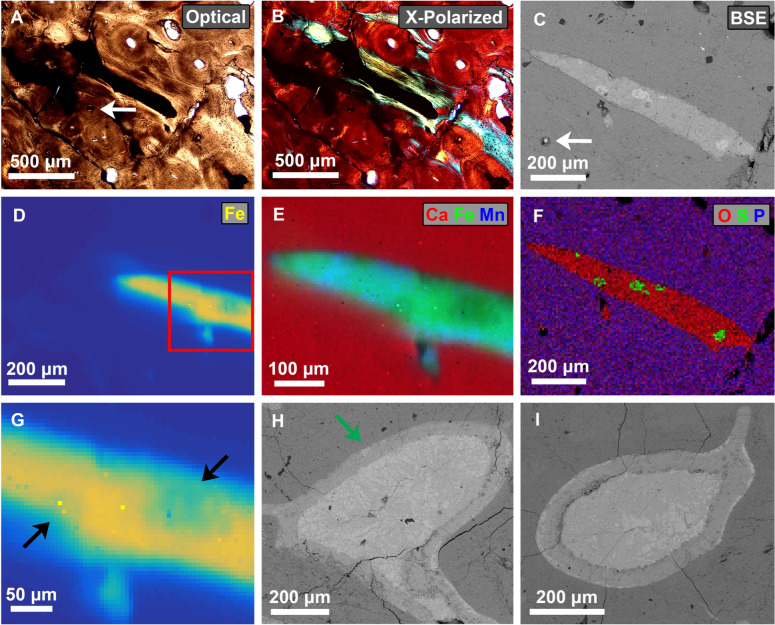
Multi-technique imaging of the exposed vessel-like structure surface from RSKM P2523.8. (**A**–**G**) Analysis of the vessel-like structure corresponding to Fig. 3B–D (red arrow). (**A**) Optical and (**B**) cross-polarized light microscopy of the structure using a thin section slide produced from the bone section of Fig. 1B,C. The structures appears completely opaque compared to the surrounding bone matrix. In this orientation original bone osteons are above the vessel, and callus bone osteons are below the vessel. (**C**, **F**) SEM analysis of the bone section of Fig. 1B,C, with white arrows showing correspondence to the same Haversian canal. (**C**) Back scattered electron image of the structure, showing brighter areas that have higher atomic number composition. (**F**) RGB map showing the relative amounts of oxygen, sulphur, and phosphorus in the structure. (**D**, **E**, **G**) VESPERS beamline chemical mapping at CLS. Pixel resolution = 5 $\upmu$m. (**E**) Calcium, iron, and manganese RGB map of the structure. (**D**) Iron concentration map of the structure, the most dominant element. (**G**) Zoom of (**D**) showing the heterogeneity of the iron in the structure, with areas of reduced iron shown. (**H**) BSE image of another vessel-like structure with 3DCT correspondence to Fig. 3C,D (green arrow), featuring a distinct outer layering. (**I**) BSE image of a structure in Zone 1 with similar morphology to (**H**), but with no 3D correspondence.

The “blood vessel-like structures” found in Fig. 3 (herein referred to as “vessels” for brevity) were also imaged using further higher resolution techniques in order to provide an examination of their morphology and to determine their general chemical composition (Fig. 4). Normal transmitted and cross polarized light microscope images of a thin section of the vessels with 3DCT correspondence (Fig. 3B–D, red arrow) show that this area is highly opaque to visible light compared to the surrounding bone matrix (Fig. 4A,B).

SEM analysis was performed, where the back scattering electron image of this vessel shows that its composition is distinct from the surrounding bone (Fig. 4C). It is fairly uniform in atomic number except for some small patches of higher atomic numbers throughout (brighter areas correspond to higher atomic numbers). EDS mapping of the entire region was also performed (Fig. 4F), which was useful for probing low transition energy elements (<4 MeV) such as carbon and oxygen. This RGB map gives the relative concentration of the most abundant elements in the sample, so they can be viewed on the same plot. Phosphorus dominates outside the vessel within the regular bone matrix. Oxygen corresponds to most of the vessel, and sulphur corresponds to the higher atomic number patches found in the BSE image (Fig. 4C). Low statistics from other elements probed in the EDS analysis demonstrates that these are not significant within the vessel. While the carbon content was high, it was uniform throughout the exposed vessel and bone area, suggesting the carbon found is entirely the result of the conductive carbon coating commonly used in SEM analysis, and therefore it is unlikely that endogenous carbon is present in the vessel. All SEM-EDS chemical maps for all relevant elements are given in the supplementary information (Supplementary Fig. S2), which also shows presence of iron and silicon in the vessel.

Further chemical imaging at the CLS VESPERS beam line allowed probing of higher transition energy elements, such as strontium and yttrium. Here, the most dominant elements in the scan are calcium, iron and manganese, represented in an RGB map (Fig. 4E). Calcium, as expected, dominates the bone matrix, while the vessels are dominated by iron with lesser contribution due to manganese. Visualizing the iron distribution only (Fig. 4D) shows other surrounding mineral inclusions in the bone also incorporate iron, but are multiple orders of magnitude less concentrated (Supplementary Fig. S4). Looking closer at the iron distribution in the vessels (Fig. 4G) shows some areas of heterogeneity, where iron concentration is notably reduced. These areas of lower iron content appear to correspond to the location of bright patches on the corresponding BSE image (Fig. 4C). There are also traces of the elements lead, nickel, and zinc (Supplementary Figs. S3, S4). All chemical maps from VESPERS are given in the supplementary information (Supplementary Fig. S4). Imaging analyses using another CLS beam line, SXRMB, was also performed (Supplementary Fig. S5). This allowed probing of elements from sulphur to iron. This also confirms the large concentration of iron in the vessels and sulphur infusions shown in the BSE image (Fig. 4C), as well as the presence of traces of manganese and potassium.

Another exposed section of the vessel that was modeled in 3D (Fig. 3C,D (green arrow)) was scanned using the SEM (Fig. 4H). This section is much larger, and has a distinct outer border that is $\approx$50 $\upmu$m in width. Similar morphology is also found throughout Zone 1 of the bone, such as in Fig. 4I. However, we are unable to correlate these with the 3DCT models, and thus we cannot make any statements whether these are or are not angiogenic vessels.

### Chemical microprobing

**Fig. 5:**
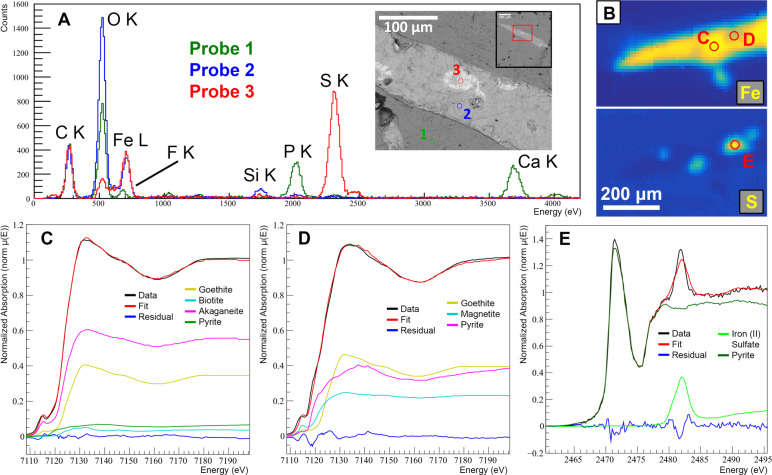
Chemical microprobe analysis of the exposed vessel-like structure corresponding to Fig. 3B–D (red arrow) and Fig. 4A–G from RSKM P2523.8. (**A**) SEM-EDS analysis for three mircoprobing locations as shown on a BSE image of the structure. Probe 1: bone matrix; Probe 2: Low atomic number vessel; Probe 3: high atomic number vessel. (**B**–**E**) Analysis using SXRMB at CLS. (**B**) Chemical map of iron and sulphur k$\alpha$ showing spot microprobing location for XANES measurements. Pixel resolution = 10 $\upmu$m. (**C**) Iron K-edge LCF of the main composition of structure ($\chi ^2 = 0.00873$). (**D**) Iron K-edge LCF of the high atomic number area of the structure ($\chi ^2 = 0.03736$). (**E**) Sulphur K-edge LCF the high atomic number area of the structure ($\chi ^2 = 0.12108$). Full XANES statistics are given in Supplementary Table S4. Reference spectra used in the fitting are shown in Supplementary Fig. S8.

Specific EDS spot measurements were taken with longer live measurement times on the vessel shown in Fig. 4A–G (Fig. 5A). Outside of the vessel is normal bone matrix (Fig. 5A, probe 1). The presence of calcium, phosphorus, oxygen and fluorine suggests bone hydroxyapatite ($\hbox {Ca}_5 (\hbox {PO}_4)_3(\hbox {OH})$) that has been at least partially replaced by fluorine ions to form fluorapatite ($\hbox {Ca}_5 (\hbox {PO}_4)_3 \hbox {F}$). The main composition of the vessel (Fig. 5A, probe 2) is dominated by oxygen and iron, suggesting presence of iron oxide. There is also a small contribution due to silicon, which could also be the result of silica ($\hbox {Si} \hbox {O}_2$). Throughout the vessel there are also small patches of high atomic number material (Fig. 5A, probe 3) with high contribution of iron and sulphur, suggesting the presence of pyrite ($\hbox {FeS}_2$). Spot microprobing was also performed on the features of Fig. 4H,I (Supplementary Fig. S6,S7). The results show the border composition is the same as the main composition of the vessel of (Fig. 5A), while the interiors larger than 100 $\upmu$m have a higher atomic number infill (Supplementary Figs. S6, S7). While having very similar composition compared to the border, the infill has roughly half the amount of silicon, possibly explaining the shade difference in the BSE image (Fig. 4C). Quantitative statistical analysis of the EDS spectra was produced from the SEM software (Supplementary Table S1). However, the uncertainty in the values of atomic weight percentages means a more sensitive method is required in order to provide a robust quantitative molecular analysis of the vessels.

At SXRMB, XRF mapping and XANES micro-probe analyses were performed on multiple areas of the exposed vessels that had been imaged and chemically mapped with other techniques (Fig. 5B–E). As the vessels feature high overall concentrations of iron compared to other probed elements (Fig. 5B), it was the element of focus for XANES microprobing. Sulphur was chosen as well, because it has high concentration in particular spots on the vessel (corresponding to the bright areas on the BSE image (Fig. 4C). The reference spectra used for the XANES Linear Combination Fitting are given in Supplementary Fig. S8. XANES combinatorial LCF analysis of the iron K-edge suggests the iron in the vessel is composed predominantly of iron(III) oxide-hydroxide (FeO(OH)) (95.6 ± 0.4 %, Supplementary Information), with the specific polymorphic form unknown, but likely goethite ($\alpha$ form). The best fit (Fig. 5C) suggests other possible small contributions may come from pyrite ($\hbox {FeS}_2$). XANES LCF of the sulphur K-edge on these hot spots (Fig. 5E) show the sulphur there is in the form of pyrite S$^{1-}$ ($\hbox {FeS}_2$) ($\sim$88%), with small amounts of S$^{6+}$ such as in iron sulfate ($\hbox {FeSO}_4$). The iron K-edge was also probed at the same location of the sulphur hot spot (Fig. 5D), and this region appears to be composed of a mix of equal amounts of goethite (40.4 ± 0.9 %) and pyrite (36.3 ± 1.7 %), as well as magnetite (23.3 ± 1.8 %) ($\hbox {Fe}^{2+}\hbox {Fe}^{3+}_2\hbox {O}_4$). This high atomic number region of the vessel appears to have roughly equal amounts of both iron(III) and iron(II). Full fitting statistics for the LCF are given in Supplementary Table S4. A mineral inclusion near the vessel was also probed using XANES (Supplementary Fig. S9). This vessel has nearly two orders of magnitude lower iron concentration, and the Fe K-edge LCF shows the iron form is notably different from the two probes of Fig. 5C,D, containing goethite as a dominant component, but with substantial concentration ($\sim$25%) both to hematite ($\alpha$-$\hbox {Fe}_2\hbox {O}_3$) and siderite ($\hbox {FeCO}_3$), leaving the iron(III) content at approximately two-thirds.

## Discussion

### Summary of results

The apparent vascular structures (vessels) in the fractured rib from RSKM P2523.8 were analyzed with complementary techniques using visible light, electrons, and synchrotron X-rays such that the taphonomic pathway of preservation can be inferred. A summary of the results from all techniques is given in Table 1.

**Table 1:** A summary of the results in the analysis of specimen RSKM P2523.8 highlighting the observed differences between bone and vessel structure. (HC - Haversian canals; VC - Volkmann Canal).

Technique	Reference	Bone	Vessels
2-D Histology	Fig. 1	Zone 1—edge of medullar bone: crushed osteons and HC; zone of intense reworking and remodeling. Zone 2—outer edge of cortical bone: closely packed HC; well-developed primary and secondary osteons; preserved VC. Zone 3—callus: separated by a fracture; bone changes direction of growth	Haversian and Volkmann’s canal (HC and VC) and larger angiogenic-like structures (preserved in two generations/layers)
Petrographic microscopy	Fig. 2A–C, Fig. S1	Zone 1—heavily overprinted by Fe-rich minerals; Zone 2,3—relatively limited overprinting by Fe-rich minerals	Locally, HC exhibit 2 different generations of Fe-rich mineralization; VC completely infilled; HC from open to completely infilled
Reflective microscopy	Fig. 2D–F	Primary mineralogy hydroxyapatite $\hbox {Ca}_5 (\hbox {PO}_4)_3(\hbox {OH})$ to fluorapatite $\hbox {Ca}_5 (\hbox {PO}_4)_3 \hbox {F}$	Two generations of pyrite: 1st—fine, botryoidal; 2nd—coarse-grained; Pyrite is likely partially oxidized to hematite or goethite; Galena, pyrrhotite, magnetite also observed
Synchrotron Tomography (3D virtual histology)	Fig. 3, Morphosource^43^	Uniform Density	Long, meandering, high-density structures, orthogonal to the osteons, from Zone 2 close to callous, 100–500 $\upmu$m; Even larger (up to 1 mm) vessel-like structure in Zone 1
SEM BSE imaging	Fig. 4C,H,I, Fig. S6,7	Uniform Density	Vascular infills have two distinct layers: first layer is 50 $\upmu$m in radius, second layer fills in larger cavities.
SEM EDS	Figs. 4F, 5A, Figs. S2, S6, S7, Tables S1–S3	Elements: Ca, P, O; Trace amounts: F	Elements: Fe, O with patches of S; Trace amounts: Si
Synchrotron XRF: VESPERS (Beamline 1)	Fig. 4D,E,G, Figs. S3, S4	Elements: Ca, P; Trace amounts: Sr, Y	Elements: Fe dominant with patches of Pb, Zn, Ni; Trace amounts: Mn, Ba
Synchrotron XRF: SXRMB (Beamline 2)	Fig. 5B, Fig. S5	Elements: Ca, P	Elements: Fe dominant with patches of S; Trace amounts: Mn, K
Synchrotron XANES	Fig. 5C-E, Fig. S9, Table S4	*Future Studies*	Layer 1 of vessel canals: FeOOH $95.6 \pm 0.4$ % of bulk Fe; Strong S contribution in certain parts in form of pyrite (FeS_2_) (∼88%); Fitting also suggests contribution of magnetite or hematite

Petrographically (Fig. 2), the studied bone displays well-preserved bone microstructure showing vascular canals, primary and secondary osteons and pore spaces coated or infilled by Fe-rich minerals. After the bone was buried, the existing fractures, like the one that was in the process of healing, but also the new ones due to compaction and deformation, were also completely infilled by Fe-rich minerals. Reflected microscope analysis suggests the composition has been oxidized to a mineral such as hematite. The bone also seems to display two generations of fractures, one not fully cemented, but rather having just a coating of pyrite oxidized, produced by the contact with an oxygen-rich environment. The partially filled fracture marks the separation between the callus and the underlying bone, and further fractures appear after initial burial and compaction. These open fractures are later taphonomic processes affecting the bone and indicate prolonged shallow burial or subaerial exposure during exhumation events. With the bone remaining near the surface, some of the fractures were reactivated and in some of the Haversian canals and pores gypsum and/or anhydrite was precipitated.

Initial analysis of the SR-$\upmu$CT scans (Fig. 3) show the presence of large, meandering, high-density branching structures connecting central, remodeled bone matrix to callused bone that has formed on the side of a fractured rib head from RSKM P2523.8, the Saskatchewan *T. rex*. This suggests these structures could be representative of fracture-induced angiogenic blood vessels^19^ with 3D morphology similar to results from experiments on mice^42^. If these are blood vessel structures, it appears that they are likely preserved as permineralized casts that have been partially filled in. In situ, high resolution, 3D dinosaur angiogenesis has not been reported before in literature.

Three complementary but independent methods of measurement were used to describe the quantitative chemical composition of the high-density vessels, using electron microscopy and two different micro probe beamlines at the Canadian Light Source (Figs. 4,5). Using all three methods, these vessels are shown to be dominated by iron. Using XANES, this iron is shown to be in the 3+ state corresponding predominately to some form of iron(III) oxide-hydroxide (goethite or akaganeite), the same molecules associated with other dinosaur blood vessel studies based on smaller structures^8,15^. For an iron K-edge measurement on the point of highest iron concentration, the iron(III) oxide-hydroxide contribution is $95.6 \pm 0.4$ % (Fig. 5C, Supplementary Information). This composition is fairly uniform throughout the vessel observed, with the exception of small isolated patches of pyrite ($\hbox {FeS}_2$). On the larger diameter vessels, there is a distinct (first) outer depositional layer with a width of 50 $\upmu$m that appears to have a similar composition to the smaller vessels, but also contains a slightly higher atomic number interior. Other notable elements found in the vessels are manganese, with trace amounts of the elements nickel, lead, zinc, potassium, and silicon. Throughout the analysis no indications of organic preservation were found, as carbon was not present in the vessels. These chemical results also support the idea that these supposed vessels have been preserved as permineralized casts, and that the angiogenic blood vessel casts and Haversian/Volkmann’s canals share a similar composition.

Combining all the techniques allows a thorough description of the mechanisms that allowed preservation of the vessels within the rib. Petrographic analysis shows pyrite of different types, and the BSE images of Fig. 4 show that there are two distinguishable minerals, the second layer being higher Z. Pyrite is also more likely to be oxidized to goethite when in silica rich soils^44^, and more silica is seen in the first deposition layer compared to the second via the EDS analysis (Supplementary Figs. S6, S7). Therefore we conclude that the inner (second) layer is likely a different iron oxide to geothite, such as hematite. Synchrotron analysis focused on Zone 2, where the large angiogenic vessels are located. More synchrotron analysis of the Zone 1 and 3 vessels will be performed in a future experiment to compare the iron forms in all zones, and to provide quantitative identification of the molecular form of iron in the second deposition layer. To summarize the composition of the vessel casts (Supplementary Fig. S10), canals less than $100$ $\upmu$m in diameter are filled with fine-grained (botryoidal) pyrite which oxidized to goethite FeO(OH) almost completely. Larger canals are filled in with an interior second layer of crystalline pyrite that has only partially been oxidized to an iron oxide that differs from goethite, likely hematite ($\hbox {Fe}_2\hbox {O}_3$). A layer of “rust” in between which will be some mix of hydrous FeO(OH) and $\hbox {Fe}_2\hbox {O}_3$.

### Analysis

During diagenesis, some of the most common, yet very complex, processes are precipitation and mineral replacement infilling porosity available in the bone, such as vascular canals^45–49^. The dinosaur bone studied showed a similar path to Previtera^50^ (Supplementary Fig. S11), but with some different cements being precipitated. In summary, the taphonomic path of the studied bone could be described as death, exposure to the environmental conditions causing fracturing and flaking, burial causing further compression, permineralization and fracturing, prolonged shallow burial or cyclical exhumation and reburial, and then the final deeper burial. Initially, the vascular canals and other pore spaces, such as fractures, were coated by fine-grained pyrite, indicating that the permineralization took place in an anoxic environment, in the presence of decaying of organic matter in the sediment and likely the presence of sulphate in solution in the pore water. The bone and the pyrite subsequently came in contact with oxygen, either through exhumation or changing conditions in the pore spaces within the surrounding sediment as water levels fluctuated. The reaction between the pyrite, water and oxygen created the iron oxide/hydroxide layer (the rust). During arid periods, in the absence of water, the pyrite would have reacted with the oxygen, at low temperatures and acidic pH environment, creating the goethite^44,51^. The bone would have been submerged in anoxic waters again to allow for the second generation of pyrite to precipitate. This second generation of pyrite has a crystalline form indicating a higher temperature or a higher degree of saturation in the fluid^52^. Finally, during the last exhumation event, the pyrite, in contact with a neutral pH, oxygen-rich environment started oxidizing forming hematite^53^, but the process was not completed. Anhydrite and/or gypsum were the last cements to precipitate, but they are very minor. This suggestion of a brief period of acidic environment before being regulated by ground water to a more neutral pH soil conditions, would have allowed the preservation of the bone apatite and goethite long-term^8^. The occurrence of two intervals of mineralization may also help explain how plant fossils are also found in conjunction with the RSKM P2523.8 bone deposit, which generally require opposing chemical environments^32^.

For the preservation of the vessel casts, the origins of the iron required could be speculated. As conventionally soft tissue preservation is a result of mineralized replacement of original organic molecules, a first assertion is that the RSKM P2523.8 rib vessel casts were preserved in this way. The presence of fluorine, strontium, and barium that are known diagenetic replacements of hydroxide in the bone apatite are observed in the bone matrix of the RSKM P2523.8 rib, which suggests sufficient ion transfer through pore water into the bone. If there was sufficient available iron(III) in the burial pore waters that the bones endured, this could explain the presence of iron in the mineralized vessel casts. Large, angiogenic vessels provide much larger surface/volume for minerals to infiltrate, or to be deposited in successive layers. Another possibility is that the iron found in the vessel casts is partially sourced from the original contents of the blood vessels. Blood is composed of a majority component hemoglobin, an iron containing protein. Most evidence suggests that iron in hemoglobin is thought to be in the 3+ state^54^. Since the angiogenic vessels here are much larger than Haversian system vessels, this means more locally available iron(III) that could form goethite. In other claims of blood vessel preservation in dinosaurs^8,15^ goethite was found in the vessels, and it was suggested to be of endogenous origin. The results here partially support the limited literature on preservation of dinosaur blood vessels, but also leaves some unanswered questions that suggests there is an altered preservation pathway operating that involves the pathology.

We envisage that the fracture on the rib of RSKM P2523.8 contributes to the preservation of vessel casts described here. Since the fracture was incompletely healed, there was a higher chance of finding angiogenic vessels. We further make a postulate that pathologies, in particular due to trauma to bones, may provide a target for future soft tissue preservation searches in bone. Analysis of more samples will be needed to test this postulate, and in particular, a priority for future tests could be larger specimens. Larger bones yield larger possible fractures, leading to denser angiogenic vessel activity as needed to supply nutrients for healing, such as the case with RSKM P2523.8. This increased density would provide more chances to sample preserved vessel casts, and possibly even organic or pliable-type vessels, which will be examined in future studies. Further chemical analysis using XRF and XANES could be performed to determine if the taphonomic pathways are similar or different from those seen in RSKM P2523.8, such as testing to see if the observed dual layer deposition pattern of the vessel casts is present.

Angiogenic blood vessel casts make ideal targets for CT scanning and chemical mapping because they are large features on a backdrop of denser bone or bone with a different texture (i.e., easier to differentiate than medullary vessels and cancellous bone). While subsequent chemical analysis of the vessels was destructive in that the bone needed to be cut, the vessels within the cut slice remain in situ such that 3D CT imaging and 2D imaging and chemical analysis could be directly correlated. In other blood vessel studies, the bone was dissolved using acid in order to recover the mineralized vessels, meaning information on the location of the vessels relative to the bone is lost^8,12,14^. However, these studies found dinosaur vessels that appeared still pliable and with remnant proteins, which is something that cannot be tested at the current time with the vessel casts seen in the rib of RSKM P2523.8. Although, the combination of experimental results of this study suggest it is unlikely there is flexible organic material in the rib of RSKM P2523.8. Other dinosaur vessels are not reported to be of pathogenic origin, and are much smaller (10’s of $\upmu$m) such that they are likely part of the system of Haversian canals^8^. Recent studies^14^ used multiple imaging techniques (SEM, TEM, tabletop CT) to view layering of vessels and focused more on morphological interpretation. Use of 3D synchrotron tomography compared to tabletop X-ray systems was particularly critical in this study as synchrotron X-rays have sufficient flux to produce 3D models of angiogenic vessel casts inside large and dense fossil bones. It provides information that is both in situ and high resolution, however it appears to be underutilized in literature.

While this pilot study provides some insights about the taphonomic pathway for the fractured rib of RSKM P2523.8, it also demonstrates the potential for a much larger study that tracks the bone healing/growth potential of other dinosaurs in the Cretaceous period. By examining 3D angiogenesis of different species, ages and sizes of specimens, as well as assessing different types of pathologies (fractures and varying diseases) using various synchrotron radiation techniques, one could produce a much more comprehensive recreation of ancient life and pathologies. Comparing and contrasting this to angiogenic vessels of extant relatives such as avians or crocodiles through $\upmu$CT will allow a more comprehensive look at the evolution of these features in extinct species. These research questions will be explored in a future study using a similar suite of techniques used in this work. Overall, this work provides optimism for future in situ soft tissue analysis in fossil bone, which may unlock new understandings of taphonomy as well as the physiology of ancient life.

## Supplementary Information

Supplementary Information 1.

Supplementary Information 2.

Supplementary Information 3.

Supplementary Information 4.

## Data Availability

All analysis data are included in the main manuscript, supplementary information files, as well as at Morphosource for reconstructed CT data. All raw data produced in the experiment are available on request to the corresponding author.
