# Supplementary material for: In situ analysis of vascular structures in fractured Tyrannosaurus rex rib
Source: Sci Rep. 2025 Jul 4;15:20327. doi: 10.1038/s41598-025-06981-z (PMC12227628; doi:10.1038/s41598-025-06981-z)
Supplement: Supplementary file 1 — Supplementary Information 1. [file 41598_2025_6981_MOESM1_ESM.docx]

***In situ* analysis of vascular structures in fractured *Tyrannosaurus rex* rib**
Jerit L. Mitchell^1^*, Mauricio Barbi^1^, Ryan C. McKellar^23^, Monica Cliveti^4^ and Ian M. Coulson^4^
^1^Department of Physics, University of Regina, Regina, SK, S4S 0A2, Canada.
^2^Royal Saskatchewan Museum, 2445 Albert St., Regina, SK, S4P 4W7, Canada.
^3^Department of Biology, University of Regina, Regina, SK, S4S 0A2, Canada.
^4^Department of Earth Sciences, University of Regina, Regina, Saskatchewan, S4S 0A2, Canada.
*Corresponding Author: [jlm696@uregina.ca](mailto:jlm696@uregina.ca)

**Supplementary Information:**

**Tomography**

All μCT data produced in this analysis, including tomographic slice data and 3D mesh models, are freely available for viewing and download at MorphoSource:

(https://www.morphosource.org/projects/000509092)

**Chemical Mapping**

All relevant individual X-ray elemental maps of the probed vessel-like structure (Fig. 2 red arrow) and of surrounding bone were obtained using SEM-EDS (Fig. S2), CLS-SXRMB (Fig. S5), and CLS-VESPERS (Fig. S4). All these techniques are needed for optimised coverage of X-ray emission at different energy ranges (100-5000 eV, 1700-10000 eV, and 6000-30000 eV respectively), thereby allowing the concentrations of a greater range of elements to be examined.

**Additional VESPERS/XRF methodology/analysis**

Fig. S3 provides the spectral fluorescence sum of the entire region. The peaks correspond to various transitions in the sample. Large peaks can be easily identified using a fluorescence line energy database, such as Hephaestus (part of the Demeter package). If a Kα transition for an element is observed in the spectrum, other strong transitions such as Kβ would also be expected to present in the spectrum; thereby providing confirmation of the presence of that element. The sum florescence spectra shows that iron dominates the region, with the second and third most common elements being calcium and manganese, respectively. Strontium and yttrium are also present and are common in the diagenetic alterations of bone [1]. Peaks with low statistics and/or convoluted with nearby peaks can be determined by taking cuts on the data. Using this, the trace elements nickel, lead, zinc, and barium are also found. Other peaks labeled ``?" could not be identified. The peaks labeled in red are due to XRF artifacts and do not provide any new information about the elemental make up of the sample.

Creating chemical maps allows tracking specific elements within the vessel-like structure. To create chemical maps, a single peak from each pixel spectrum is integrated and used as the intensity value of each pixel on the map. Using this technique, the elemental maps for the largest nine identifiable transitions are given in Fig. S4 and the area mapped provides partial coverage of the first vessel-like structure imaged (Fig. 3 red arrow). The illustrated maps show high concentrations of iron in the structure, as well as manganese. Traces of other elements, e.g., nickel, zinc, and lead, are found in various distributions within the structure. Nickel and zinc occur as components in the main structure, while lead appears to match with areas of high Z as viewed from the BSE image. Calcium, as expected, is only present within the regular bone, and has low concentration in areas corresponding to the “empty” Haversian canals of the osteons in the bone. To the left of the main probed vessel-like structure is another mineralized structure which has a similar composition. Yttrium appears to be uniform throughout the regular bone, but strontium is more localized to the Haversian canals. Barium is correlated with strontium, but due the overlap of the Ba Lβ transition with iron escape peaks, the chemical map is predominately a map of iron.

**EDS Micro probing**

EDS probing of additional vessel-like structures was also performed (See Fig. S6 and Table S2, Fig. S7 and Table S3).

**XANES Quantitative Analysis**

Linear combination fitting of the three regions of the vessel-like structure surface was performed. See Supplementary Datasets 1-3 for the combinatorics analysis (legends are provided in this supplementary information file). χ^2^ values are calculated with no errors on data points, so very low reduced χ^2^ values (<< 1) are expected and therefore a single χ^2^ result for a fit is meaningless and should be used for comparison purposes only.

For the iron k-edge of the main body of the vessel-like structure (Fig. 5B), the top fits were dominated by iron(III) oxide hydroxide (FeOOH), in the various polymorphic forms: akageneite, goethite, and lepidocrocite (Dataset 1). The best 66 fit combinations were chosen to be averaged as they were statistically similar seen from plotting the spread of χ^2^. Therefore, the amount of FeOOH in the main body of the vessel is calculated to be 95.6 +- 0.4 %. All 66 fits feature goethite plus some combination of other FeOOH, suggesting the form in the vessel is likely goethite over other polymeric forms. The #1 best fit was plotted in the main Fig. 5.

For the iron k-edge of the high Z area of the vessel-like structure (Fig. 5B), the top 8 fits are statistically similar, and all have roughly equal combination of goethite, magnetite, plus a small amount of another reference (Dataset 2). Therefore, the fit with just the three is the best reasonable fit and was plotted in Fig. 5.

For the iron k-edge of the mineral infill close to the vessel-like structure (Fig. S9), the top fit appears statistically distinct, with stable amounts of goethite and siderite, and other possible contributions being pyrite and hematite (Dataset 3).


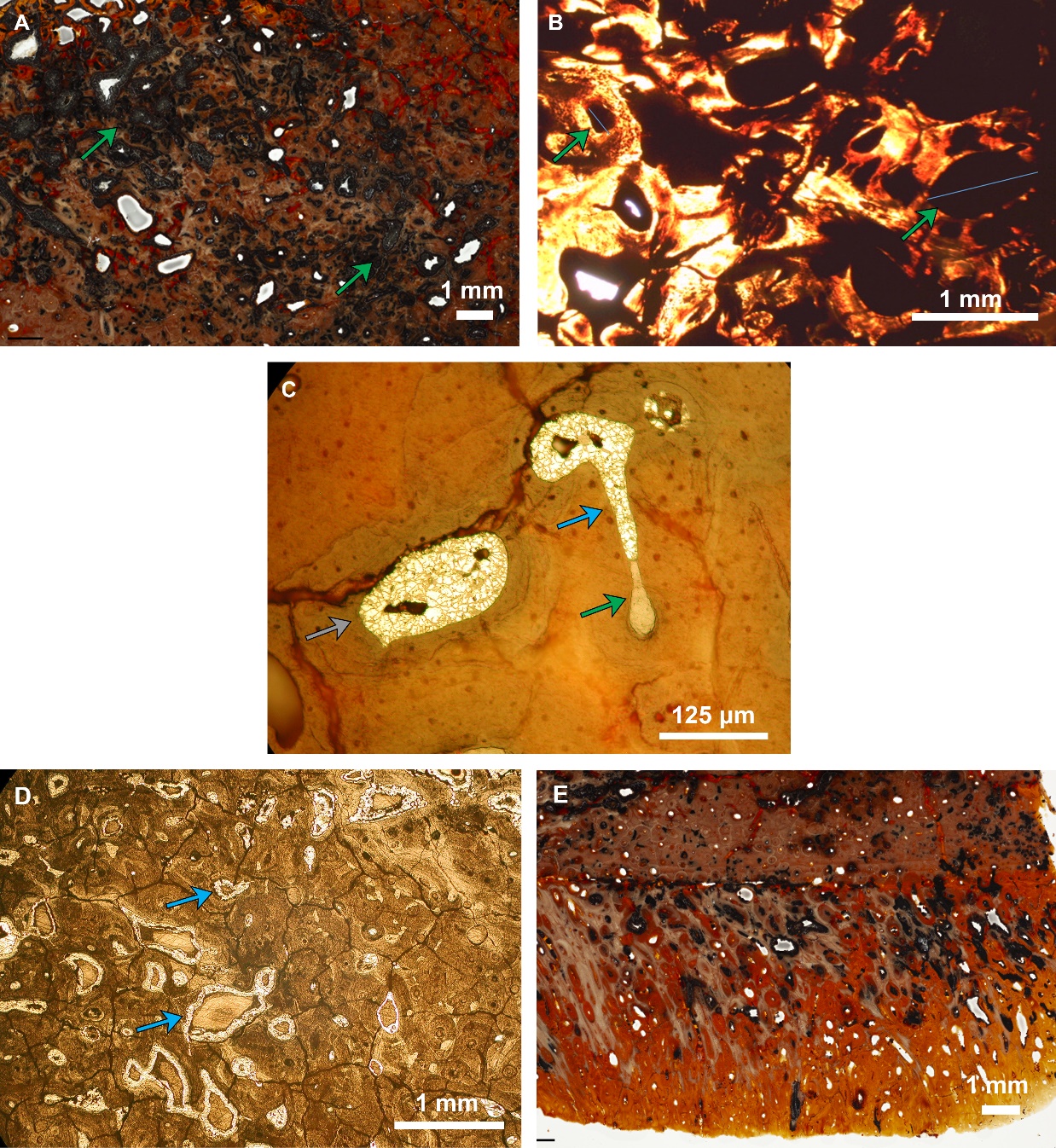


Figure S1. More Petrographic images of the fractured rib thin section from Scotty. (A) Overview of Zone 1 showing the overprinting of the initial mineralogy by Fe-rich minerals (green arrows). (B) Image in PPL, with the gypsum plate in, showing unorganized bone structure (crushed Haversian canals, Zone 1), heavily overprinted by diagenetic Fe-rich minerals. In most cases, both the Haversian canals (0.08 to 0.4mm; green arrows) and the pores are infilled. Low porosity visible by bright white areas (C) Reflective microscopy image of a Volkmann’s canal (Zone 1) showing different mineralization. The bottom part is hematite (green arrow), while the upper part is pyrite being oxidized to hematite (blue arrow). The Haversian canal to the left of it is showing the same process of pyrite being oxidized to hematite (gray arrow). (D) Overview of Zone 2 (compact bone with Haversian system) in reflected light contrast showing the coating of the Haversian canals by a generation of pyrite (mostly) (blue arrows). (E) Overview of Zone 3.


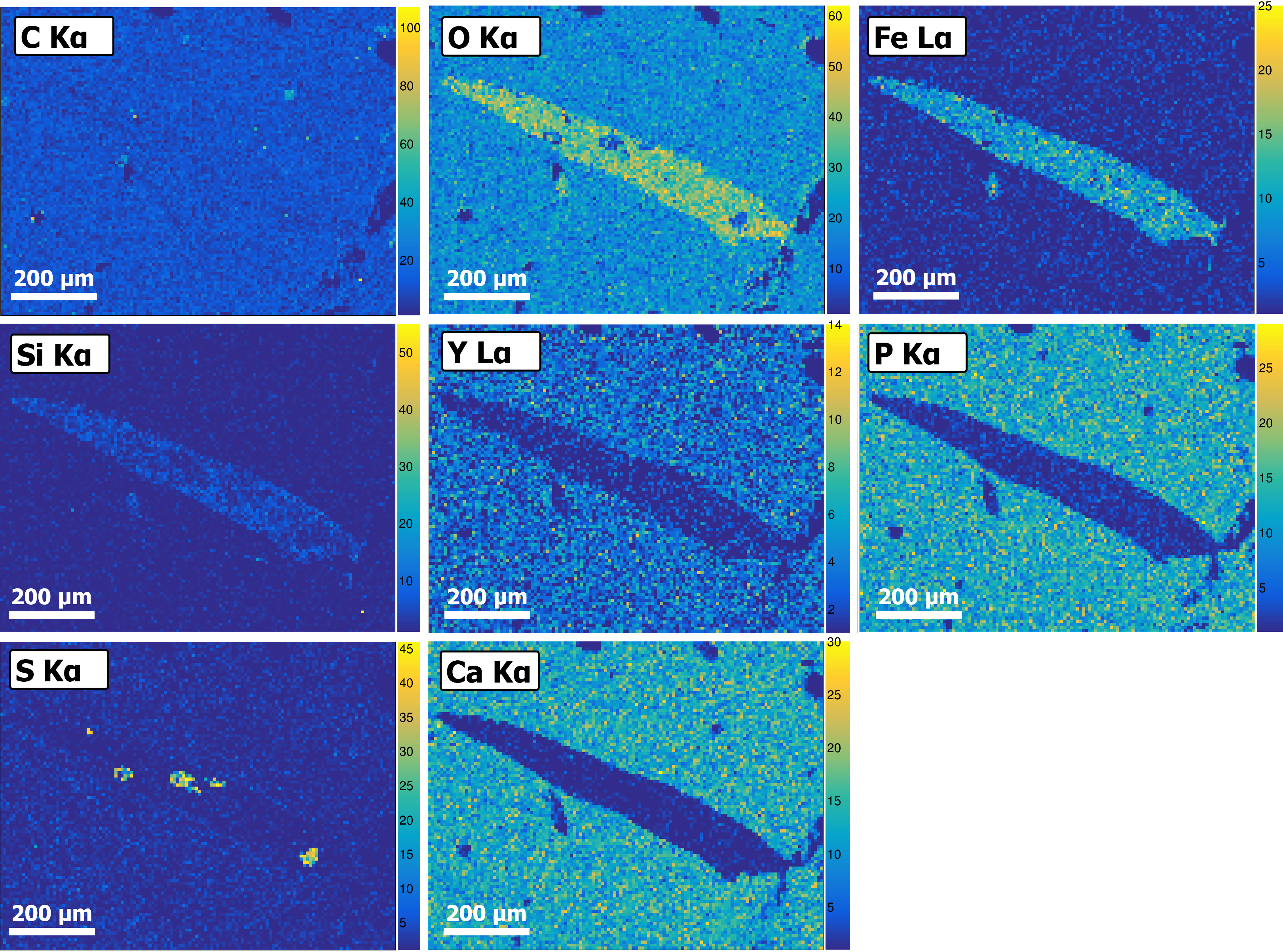


Figure S2 – All SEM-EDS chemical maps of the exposed vessel-like structure of Fig. 2B,C,D (red arrow). Raw counts for each map are provided.


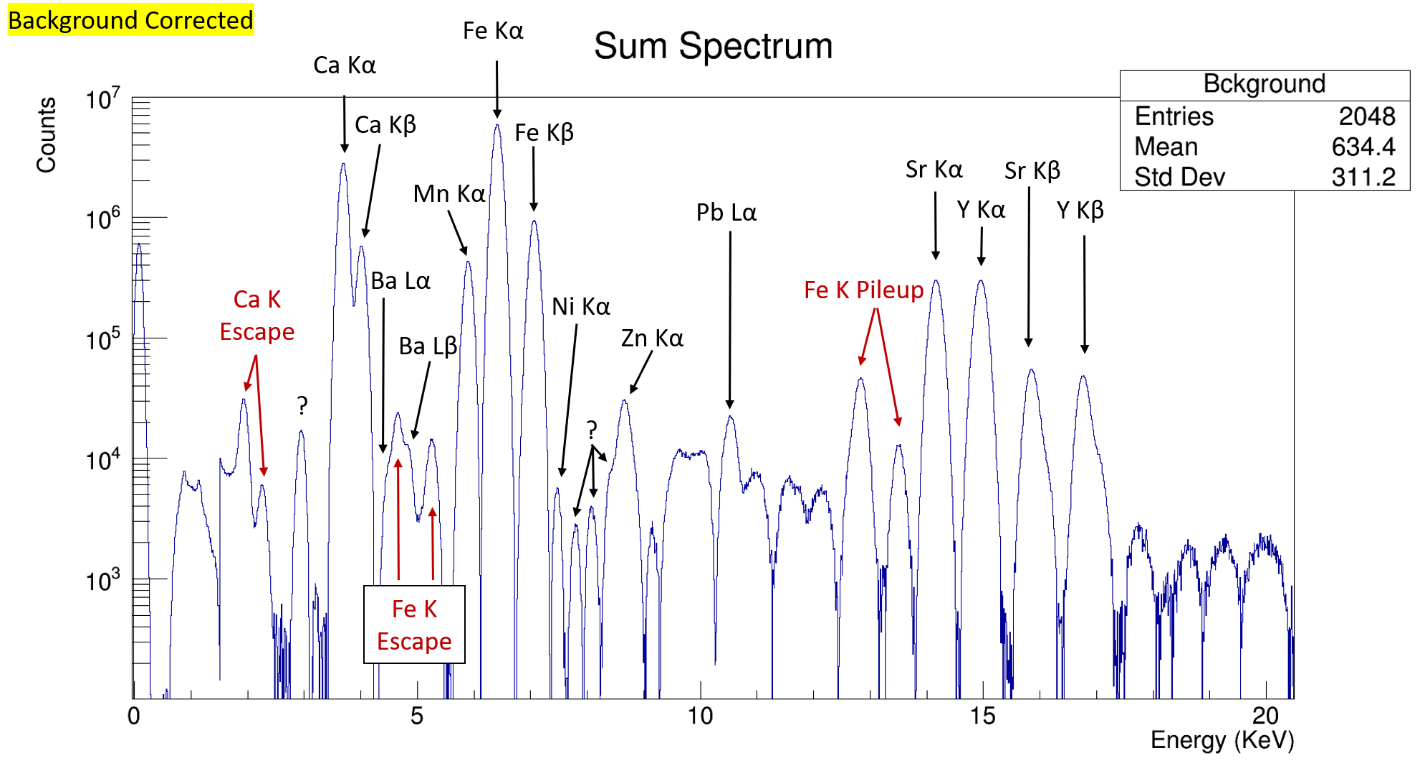


Figure S3. VESPERS XRF sum spectrum for the vessel-like structure of Fig. 2B,C,D (red arrow) which is chemically mapped in Fig. S4. Peaks labeled with black arrows are elemental transitions, while those indicated with red arrows are due to detector artifacts.


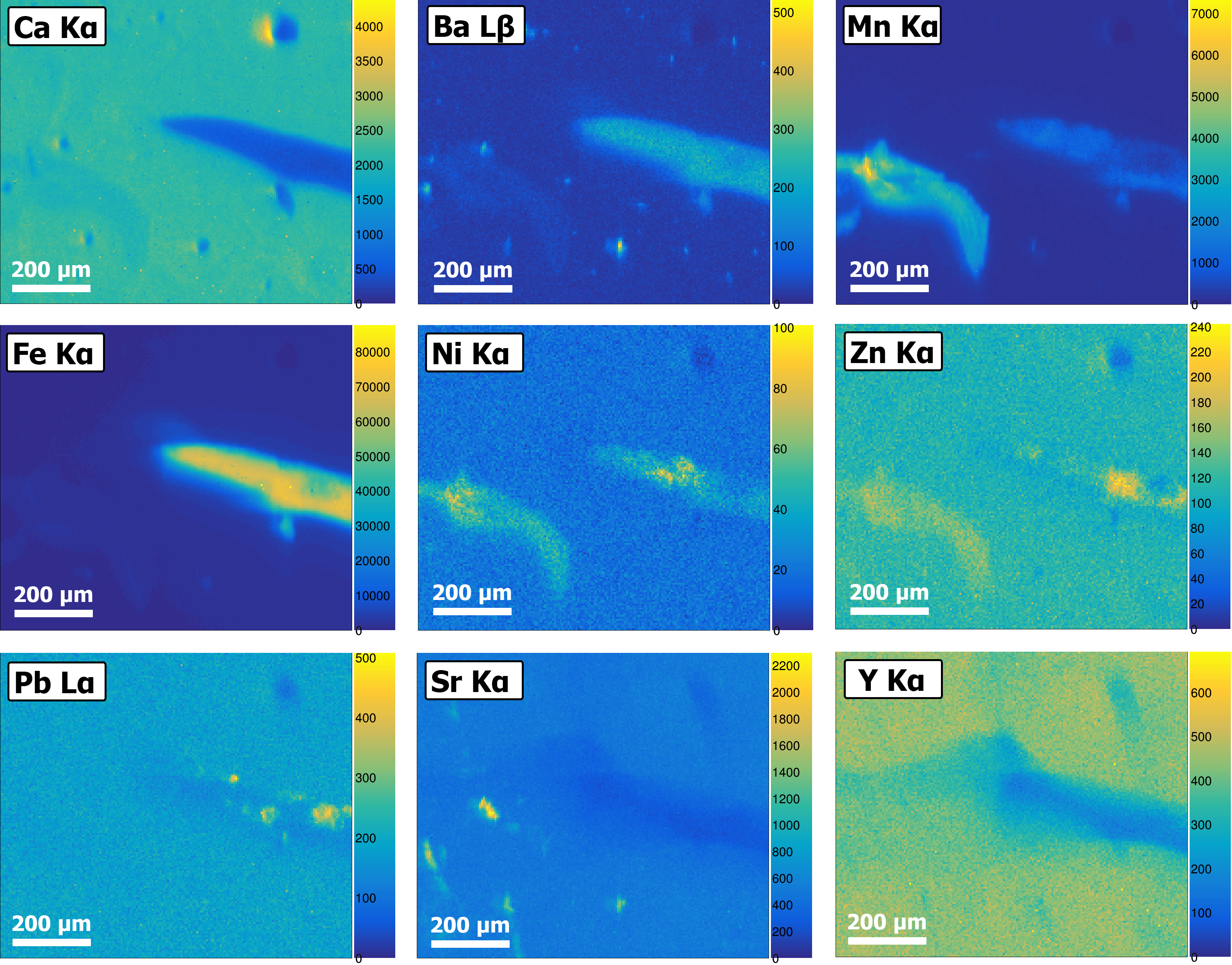


Figure S4. VESPERS chemical maps of elements found from sum spectral analysis for the exposed vessel-like structure of Fig. 2B,C,D (red arrow). Pixel size = 5 μm. Deadtime corrected count rates for each map are given in the scaling. Directly below the structure is calloused bone. Note: Due to the overlap of the barium Lβ transition with the iron Kα escape peak, the Barium map is mostly iron. When cuts are made on the strontium peak, the barium peaks are defined with less iron overlap, where barium has the apparent strontium correspondence seen.


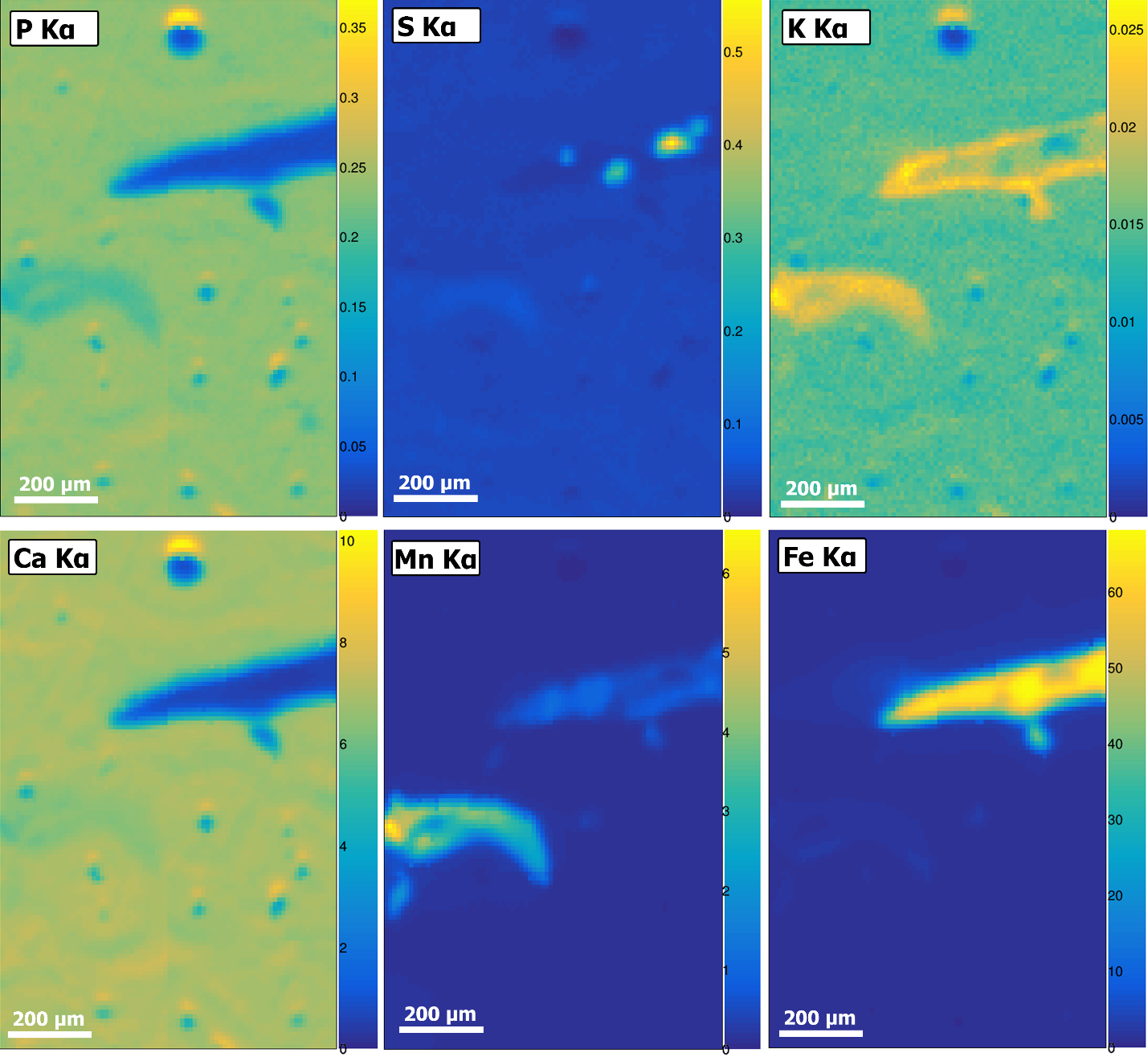


Figure S5 – All SXRMB chemical maps of the exposed vessel-like structure of Fig. 2B,C,D (red arrow). Pixel size = 10 μm. Relative counts for each map are given in the scaling. Below the structure is calloused bone.


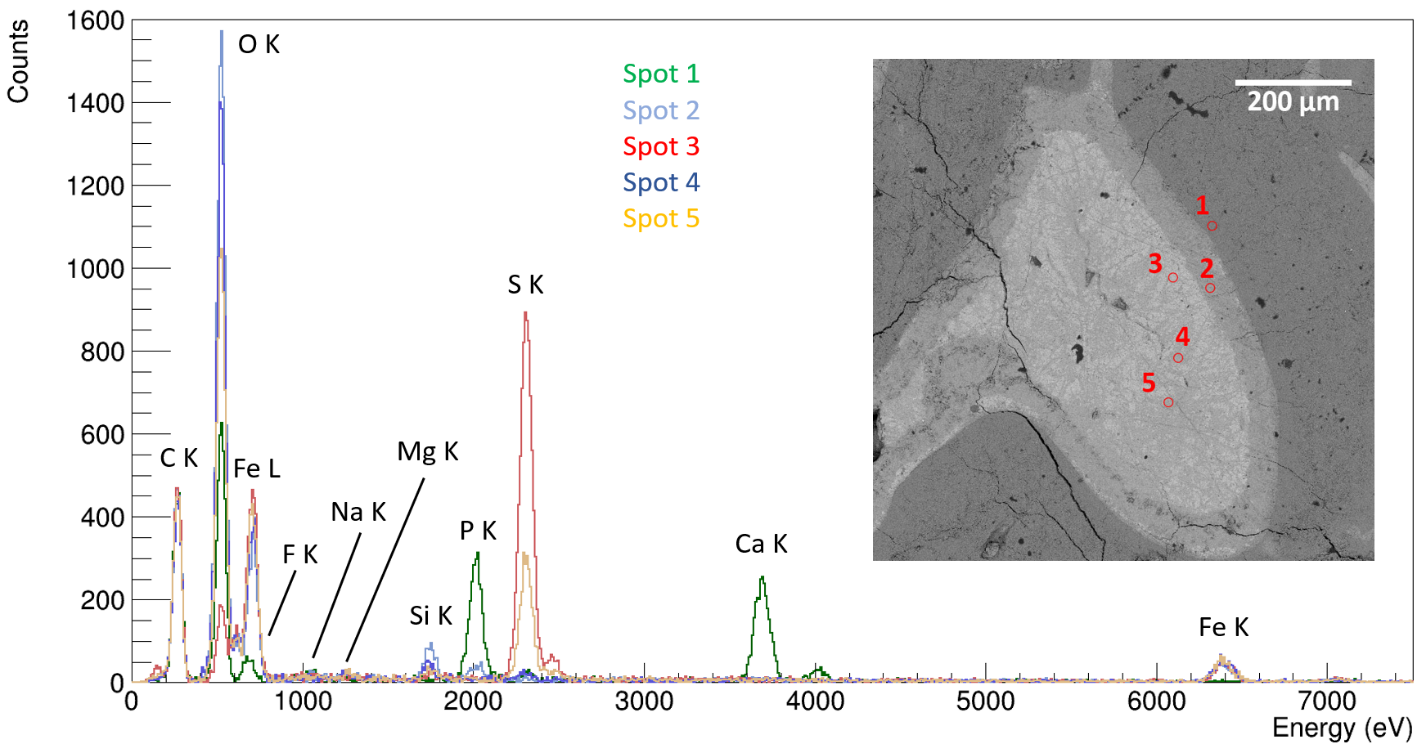


Figure S6. EDS analysis of another exposed vessel-like structure from the rib, from Fig. 2B,C (green arrow). Five probing locations as shown on a zoomed BSE image of the exposed structure. Probe 1: bone matrix; Probe 2: structure border; Probe 3: high Z structure; Probe 4: main structure; Probe 5: main structure mixed with high Z vessel.


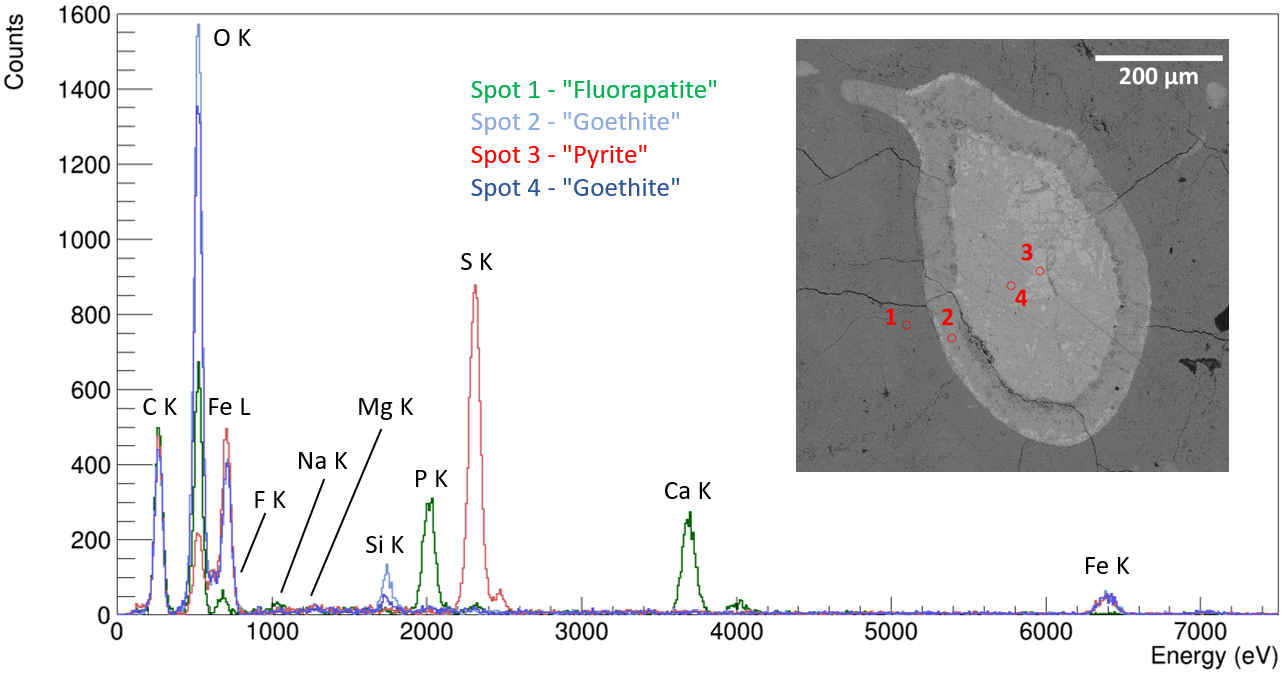


Figure S7. EDS analysis of a structure similar in morphology to S6. Four probing locations as shown on a BSE image of the structure. Probe 1: bone matrix; Probe 2: structure border; Probe 3: high Z structure; Probe 4: main structure.


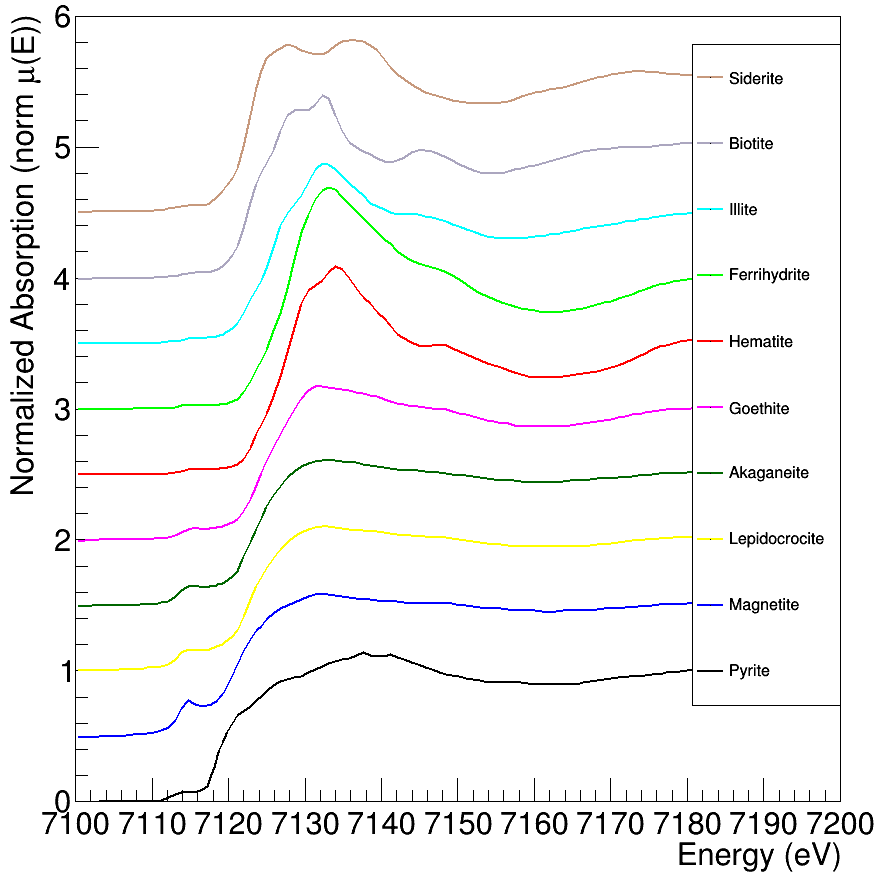

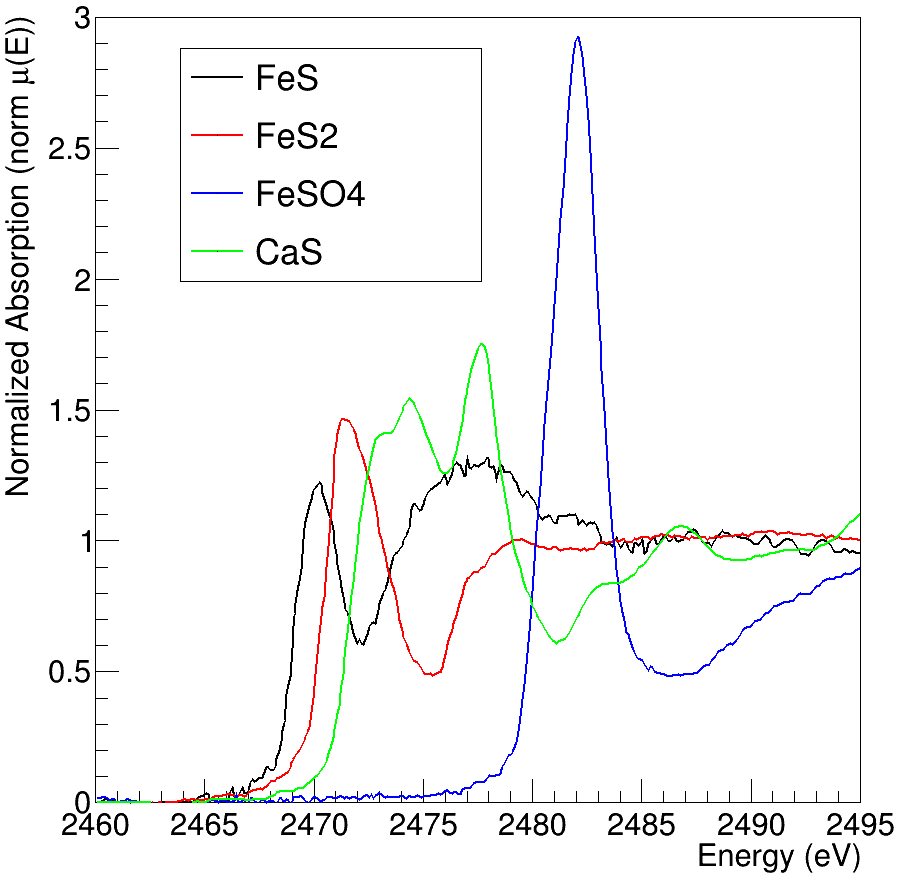
­­

……………………...(A)…………………………………………………(B)…………………….

Figure S8. XANES K-edge LCF references. (A) Ten iron mineral Fe K-edge reference spectra (sourced from [2]). (B) four sulphur and iron containing mineral references for S K-edge (sourced from the SXRMB database)


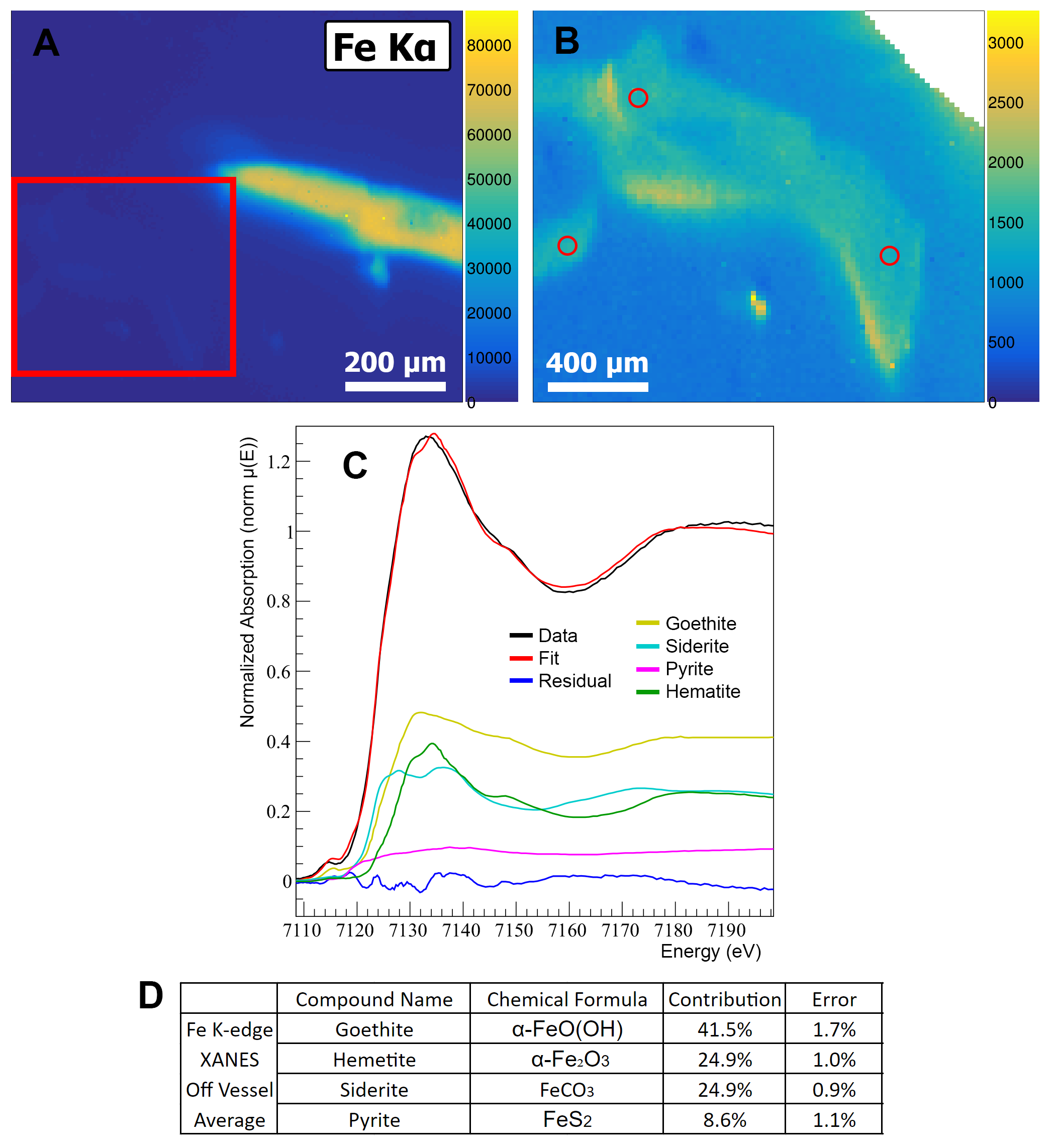


Figure S9. Chemical micro-probe analysis of the exposed surface near the corresponding vessel-like structure of Fig. 2B,C,D (red arrow) and Fig. 3A-G from RSKM_P2523.8 (Scotty). (A) VESPERS XRF iron map showing location of another iron containing structure, zoom with change in scaling seen in (B). XANES probing locations are marked in (B). (C) Iron K-edge LCF of average of the three spot measurements (χ^2^ = 0.03619). (D) Summary table of the LCF.


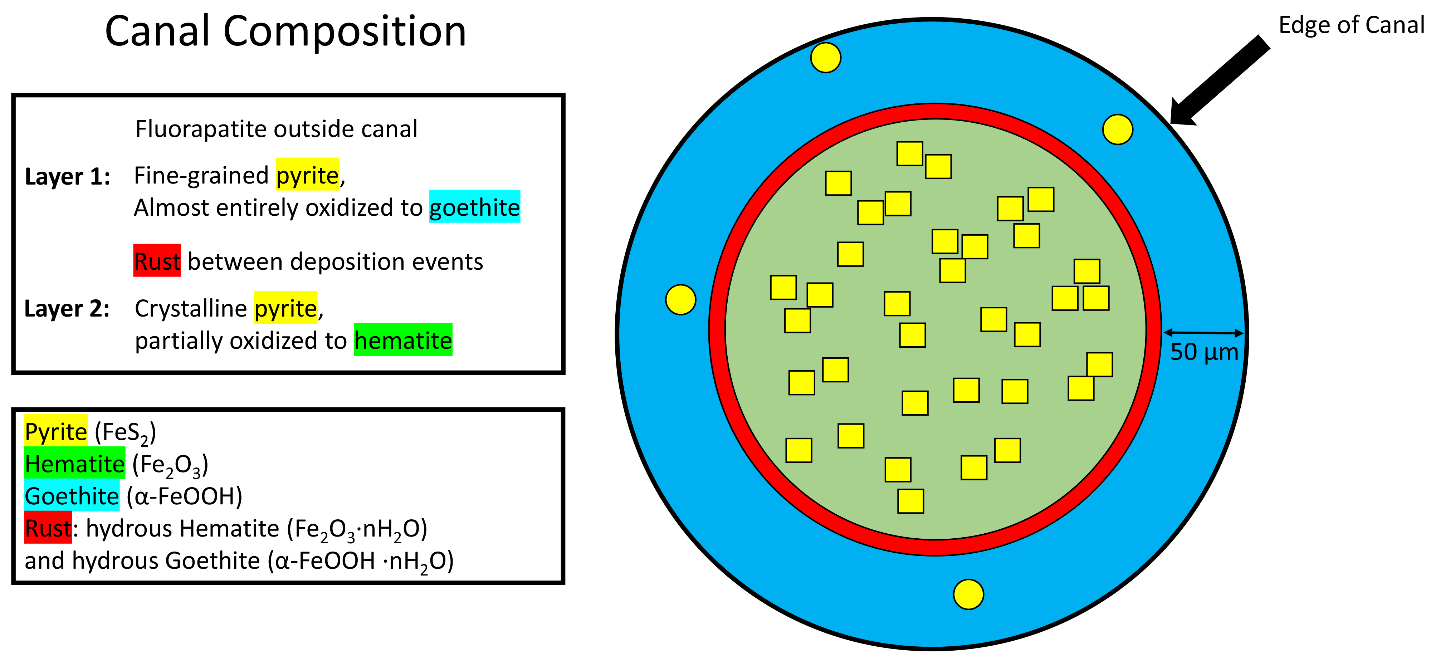


Figure S10. Simplified diagram showing the mineral layers that infill the vessel canals.


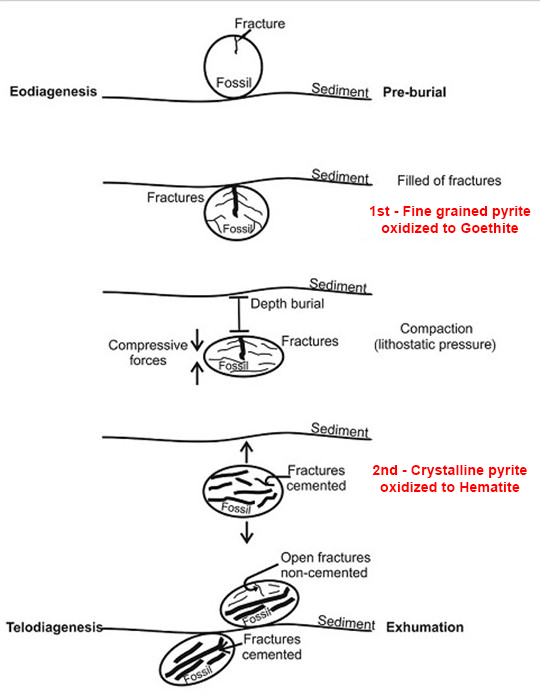


Fig S11. Suggested preservation pathway of Scotty RSKM_P2523.8. Adapted from Fig 8 of Privitera, 2017 [3]. [https://creativecommons.org/licenses/by/4.0/.](https://creativecommons.org/licenses/by/4.0/)

| **Spot 1** | **Element** | **Weight %** | **Atomic %** | **Error %** |
| --- | --- | --- | --- | --- |
|  | **C K** | 14 | 24.6 | 10.3 |
|  | **O K** | 33.6 | 44.5 | 10.5 |
|  | **F K** | 2.2 | 2.4 | 23.2 |
|  | **Na K** | 1.5 | 1.4 | 18.7 |
|  | **Mg K** | 1.2 | 1 | 15.4 |
|  | **Y L** | 2.5 | 0.6 | 14 |
|  | **P K** | 10.5 | 7.2 | 5.8 |
|  | **Ca K** | 34.6 | 18.3 | 5.5 |
| **Spot 2** | **Element** | **Weight %** | **Atomic %** | **Error %** |
|  | **C K** | 19.9 | 30.9 | 9.6 |
|  | **O K** | 48.8 | 56.8 | 6.9 |
|  | **Fe L** | 25 | 8.3 | 10.3 |
|  | **Si K** | 4.1 | 2.7 | 10.4 |
|  | **P K** | 2.1 | 1.2 | 26.8 |
| **Spot 3** | **Element** | **Weight %** | **Atomic %** | **Error %** |
|  | **C K** | 36.2 | 60.5 | 12.4 |
|  | **O K** | 5.7 | 7.2 | 15.3 |
|  | **Fe L** | 15.6 | 5.6 | 7.5 |
|  | **Si K** | 0.8 | 0.6 | 26.2 |
|  | **S K** | 41.7 | 26.1 | 3.7 |

Table S1. Semi-quantitative SEM-EDS elemental composition analysis produced from EDAX software of the main vessel-like structure area corresponding to Fig. 4. These results have large error values (from both systematic and statistical sources) and therefore no reasonable conclusions can be extracted from them.

| **Spot 1** | **Element** | **Weight %** | **Atomic %** | **Error %** |
| --- | --- | --- | --- | --- |
|  | **C K** | 16.2 | 28.5 | 10.2 |
|  | **O K** | 30.4 | 40.2 | 10.8 |
|  | **F K** | 2.7 | 3 | 19.5 |
|  | **Na K** | 1.7 | 1.5 | 17.5 |
|  | **Y L** | 2.9 | 0.7 | 13.3 |
|  | **P K** | 10.9 | 7.4 | 5.9 |
|  | **Ca K** | 35.2 | 18.6 | 5.6 |
| **Spot 2** | **Element** | **Weight %** | **Atomic %** | **Error %** |
|  | **C K** | 20.9 | 32.2 | 9.6 |
|  | **O K** | 47.4 | 54.8 | 7 |
|  | **Fe L** | 23.9 | 7.9 | 10 |
|  | **Mg K** | 1.2 | 0.9 | 23.2 |
|  | **Si K** | 4 | 2.6 | 10.5 |
|  | **P K** | 2.5 | 1.5 | 18.9 |
| **Spot 3** | **Element** | **Weight %** | **Atomic %** | **Error %** |
|  | **C K** | 35.9 | 60.7 | 12.3 |
|  | **O K** | 5.6 | 7.1 | 15.2 |
|  | **Fe L** | 17.9 | 6.5 | 7.2 |
|  | **Si K** | 0.5 | 0.4 | 31.2 |
|  | **S K** | 40.1 | 25.4 | 3.7 |
| **Spot 4** | **Element** | **Weight %** | **Atomic %** | **Error %** |
|  | **C K** | 20.1 | 31.9 | 9.1 |
|  | **O K** | 47.1 | 56 | 6.8 |
|  | **Fe L** | 30.1 | 10.3 | 9.7 |
|  | **Si K** | 2.7 | 1.8 | 12 |
| **Spot 5** | **Element** | **Weight %** | **Atomic %** | **Error %** |
|  | **C K** | 25 | 40.6 | 11.2 |
|  | **O K** | 32.9 | 40.1 | 8.5 |
|  | **Fe L** | 22.8 | 8 | 8.8 |
|  | **As L** | 1.2 | 0.3 | 32.4 |
|  | **Si K** | 1.4 | 1 | 14 |
|  | **S K** | 16.7 | 10.1 | 5.2 |

Table S2. Quantitative SEM-EDS elemental composition analysis produced from EDAX software of the other main vessel-like structure area corresponding to Fig. S6. These results have large error values (from both systematic and statistical sources) and therefore no reasonable conclusions can be extracted from it.

| **Spot 1** | **Element** | **Weight %** | **Atomic %** | **Error %** |
| --- | --- | --- | --- | --- |
|  | **C K** | 16.6 | 29.2 | 10.2 |
|  | **O K** | 30.9 | 40.7 | 10.7 |
|  | **F K** | 2.4 | 2.6 | 20.9 |
|  | **ZnL** | 1.2 | 0.4 | 32.9 |
|  | **Mg K** | 0.7 | 0.6 | 22.4 |
|  | **Y L** | 2.3 | 0.5 | 16.2 |
|  | **P K** | 11.2 | 7.6 | 5.9 |
|  | **Ca K** | 34.8 | 18.3 | 5.5 |
| **Spot 2** | **Element** | **Weight %** | **Atomic %** | **Error %** |
|  | **C K** | 19.4 | 30.3 | 9.4 |
|  | **O K** | 48.6 | 57.2 | 6.7 |
|  | **Fe L** | 26.9 | 9.1 | 9.8 |
|  | **Si K** | 5.1 | 3.4 | 7.9 |
| **Spot 3** | **Element** | **Weight %** | **Atomic %** | **Error %** |
|  | **C K** | 34.3 | 58.7 | 12.2 |
|  | **O K** | 7.2 | 9.3 | 12.6 |
|  | **Fe L** | 18.4 | 6.8 | 7 |
|  | **Co L** | 0 | 0 | 100 |
|  | **Zn L** | 0.3 | 0.1 | 65.4 |
|  | **As L** | 1.1 | 0.3 | 38.2 |
|  | **S K** | 38.7 | 24.8 | 3.7 |
| **Spot 4** | **Element** | **Weight %** | **Atomic %** | **Error %** |
|  | **C K** | 20.3 | 32.1 | 9 |
|  | **O K** | 47.2 | 56 | 6.8 |
|  | **Fe L** | 30.2 | 10.3 | 10 |
|  | **Si K** | 2.4 | 1.6 | 13.2 |

Table S3. Semi-quantitative SEM-EDS elemental composition analysis produced from EDAX software of the structure corresponding to Fig. S7. These results have large error values (from both systematic and statistical sources) and therefore no reasonable conclusions can be extracted from them.

|  | Compound Name | Chemical Formula | Contribution (%) | Error (%) |
| --- | --- | --- | --- | --- |
| Fe K-Edge Main area  (χ2 = 0.00873) (Fig. 4C) | Akaganeite | β-FeO(OH) | 55.1 | 1.6 |
|  | Goethite | α-FeO(OH) | 35.0 | 1.1 |
|  | Pyrite | FeS_2_ | 6.1 | 0.6 |
|  | Biotite | K(Mg,Fe)_3_(AlSi_3_O_10_)(F,OH)_2_ | 3.7 | 0.5 |
| Fe K-Edge High Z area  (χ2 = 0.03736) (Fig. 4D) | Goethite | α-FeO(OH) | 40.4 | 0.9 |
|  | Pyrite | FeS_2_ | 36.3 | 1.7 |
|  | Magnetite | Fe^2+^Fe^3+^_2_O_4_ | 23.3 | 1.8 |
| S K-Edge High Z area  (χ2 = 0.12108) (Fig. 4E) | Pyrite | FeS_2_ | 87.8 | 0.3 |
|  | Iron Sulfate | FeSO_4_ | 12.2 | 0.4 |

Table S4. Summary table of the best fit using LCF of XANES point measurements on the Scotty fractured rib bone from Fig. 4B-E, produced using Athena software.

**Dataset Legends**

Dataset 1: XANES Linear Combination Fitting spread sheet produced by Athena software (part of Demeter Package) for the iron k-edge of the main body of the vessel-like structure from Fig. 5B. Each individual fit performed showing weights for four possible mineral component is shown in a single row. The best fit (Fit #1) is shown in the top row, and the other rows show fits in ascending χ^2^ value. Further analysis of FeOOH was done using simple Excel calculations (to the right of raw LCF results). Graphs used for analysis include the absolute amount of FeOOH, Rolling average of 10 of FeOOH amount, and the reduced χ^2^ value plotted versus the fit number in ascending order of χ^2^ value.

Dataset 2: XANES Linear Combination Fitting spread sheet produced by Athena software (part of Demeter Package) for the iron k-edge of the high Z area of the vessel-like structure from Fig. 5B. Each individual fit performed showing weights for each mineral component is shown in a single row. The best fit (Fit #1) is shown in the top row, and the other rows show fits in ascending χ^2^ value. The summary table on the right shows the top eight fits that are nearly identical as three of their components are the same. The reduced χ^2^ plot below also shows this.

Dataset 3: XANES Linear Combination Fitting spread sheet produced by Athena software (part of Demeter Package) for the iron k-edge of the mineral infill close to the vessel-like structure (Fig. S9). Each individual fit performed showing weights for each mineral component is shown in a single row. The best fit (Fit #1) is shown in the top row, and the other rows show fits in ascending χ^2^ value. The reduced χ^2^ is plotted versus fit number in ascending order of χ^2^ value.

References

[1] Gueriau, P., Jauvion, C. & Mocuta, C. Show me your yttrium, and I will tell you who you are: Implications for fossil imaging. Palaeontology 61, 981–990, DOI: 10.1111/pala.12377 (2018).

[2] Cui, J. et al. Distribution and speciation of copper in rice (Oryza sativa L.)
from mining-impacted paddy soil: Implications for copper uptake mechanisms.
Environment International 126, 717–726 (2019).

[3] Previtera, E. Bone microstructure and diagenesis of saurischian dinosaurs from the Upper Cretaceous (Neuquén Group), Argentina. Andean Geol. 44, 39–58, DOI: 10.5027/andgeoV44n1-a03 (2017).
